# Cost-effectiveness of applying high-sensitivity troponin I to a score for cardiovascular risk prediction in asymptomatic population

**DOI:** 10.1371/journal.pone.0307468

**Published:** 2024-07-19

**Authors:** Paul Jülicher, Nataliya Makarova, Francisco Ojeda, Isabella Giusepi, Annette Peters, Barbara Thorand, Giancarlo Cesana, Torben Jørgensen, Allan Linneberg, Veikko Salomaa, Licia Iacoviello, Simona Costanzo, Stefan Söderberg, Frank Kee, Simona Giampaoli, Luigi Palmieri, Chiara Donfrancesco, Tanja Zeller, Kari Kuulasmaa, Tarja Tuovinen, Felicity Lamrock, Annette Conrads-Frank, Paolo Brambilla, Stefan Blankenberg, Uwe Siebert

**Affiliations:** 1 Medical Affairs, Core Diagnostics, Abbott, Abbott Park, IL, United States of America; 2 Midwifery Science—Health Care Research and Prevention, Institute for Health Service Research in Dermatology and Nursing (IVDP), University Medical Center Hamburg-Eppendorf, Hamburg, Germany; 3 German Center for Cardiovascular Research (DZHK), Partner Site Hamburg/Kiel/Lübeck, Hamburg, Germany; 4 Department of General and Interventional Cardiology, University Heart and Vascular Center Hamburg, Hamburg, Germany; 5 Institute of Epidemiology, German Research Center for Environmental Health, Helmholtz Zentrum München, Neuherberg, Germany; 6 German Center for Cardiovascular Research (DZHK), Partner Site Munich Heart Alliance, München, Germany; 7 Institute for Medical Information Processing, Biometry and Epidemiology—IBE, Faculty of Medicine, Ludwig-Maximilians-Universität in Munich, Munich, Germany; 8 Centro Studi Sanità Pubblica, Università Milano Bicocca, Milan, Italy; 9 Department of Public Health, Faculty of Health and Medical Science, University of Copenhagen, Copenhagen, Denmark; 10 Center for Clinical Research and Prevention, Copenhagen University Hospital–Bispebjerg and Frederiksberg, Copenhagen, Denmark; 11 Department of Clinical Medicine, Faculty of Health and Medical Sciences, University of Copenhagen, Copenhagen, Denmark; 12 Finnish Institute for Health and Welfare, Helsinki, Finland; 13 Department of Epidemiology and Prevention, IRCCS Neuromed, Pozzilli, Italy; 14 Department of Medicine and Surgery, LUM University “Giuseppe Degennaro”, Casamassima, Italy; 15 Department of Public Health and Clinical Medicine, Umeå University, Umeå, Sweden; 16 Centre for Public Health, Queen’s University of Belfast, Belfast, Northern Ireland; 17 Department of Cardiovascular, Endocrine-metabolic Diseases and Aging, Istituto Superiore di Sanità, Rome, Italy; 18 Mathematical Science Research Centre, Queen’s University Belfast, Belfast, Northern Ireland, United Kingdom; 19 Department of Public Health, Health Services Research and Health Technology Assessment, Institute of Public Health, Medical Decision Making and Health Technology Assessment, UMIT TIROL—University for Health Sciences and Technology, Hall in Tirol, Austria; 20 Department of Medicine and Surgery, University of Milano-Bicocca, Milan, Italy; 21 Center for Health Decision Science, Depts. of Epidemiology and Health Policy & Management, Harvard Chan School of Public Health, Boston, MA, United States of America; 22 Program on Cardiovascular Research, Institute for Technology Assessment and Dept. of Radiology, Massachusetts General Hospital, Harvard Medical School, Boston, MA, United States of America; University of Texas Medical Branch at Galveston, UNITED STATES OF AMERICA

## Abstract

**Introduction:**

Risk stratification scores such as the European Systematic COronary Risk Evaluation (SCORE) are used to guide individuals on cardiovascular disease (CVD) prevention. Adding high-sensitivity troponin I (hsTnI) to such risk scores has the potential to improve accuracy of CVD prediction. We investigated how applying hsTnI in addition to SCORE may impact management, outcome, and cost-effectiveness.

**Methods:**

Characteristics of 72,190 apparently healthy individuals from the Biomarker for Cardiovascular Risk Assessment in Europe (BiomarCaRE) project were included into a discrete-event simulation comparing two strategies for assessing CVD risk. The standard strategy reflecting current practice employed SCORE (SCORE); the alternative strategy involved adding hsTnI information for further stratifying SCORE risk categories (S-SCORE). Individuals were followed over ten years from baseline examination to CVD event, death or end of follow-up. The model tracked the occurrence of events and calculated direct costs of screening, prevention, and treatment from a European health system perspective. Cost-effectiveness was expressed as incremental cost-effectiveness ratio (ICER) in € per quality-adjusted life year (QALYs) gained during 10 years of follow-up. Outputs were validated against observed rates, and results were tested in deterministic and probabilistic sensitivity analyses.

**Results:**

S-SCORE yielded a change in management for 10.0% of individuals, and a reduction in CVD events (4.85% vs. 5.38%, p<0.001) and mortality (6.80% vs. 7.04%, p<0.001). S-SCORE led to 23 (95%CI: 20–26) additional event-free years and 7 (95%CI: 5–9) additional QALYs per 1,000 subjects screened, and resulted in a relative risk reduction for CVD of 9.9% (95%CI: 7.3–13.5%) with a number needed to screen to prevent one event of 183 (95%CI: 172 to 203). S-SCORE increased costs per subject by 187€ (95%CI: 177 € to 196 €), leading to an ICER of 27,440€/QALY gained. Sensitivity analysis was performed with eligibility for treatment being the most sensitive.

**Conclusion:**

Adding a person’s hsTnI value to SCORE can impact clinical decision making and eventually improves QALYs and is cost-effective compared to CVD prevention strategies using SCORE alone. Stratifying SCORE risk classes for hsTnI would likely offer cost-effective alternatives, particularly when targeting higher risk groups.

## Introduction

Cardiovascular disease (CVD) has become the single most important cause of NCD (noncommunicable disease) deaths worldwide, and it is estimated that 18 million cases, or almost one third of all deaths, are attributable to CVD [1]. In the member countries of the European Society of Cardiology (ESC), more than 109 million people are living with CVD and there are approximately 20 million new cases per year. In 2021, the management and treatment of people with CVD costs 155 billion Euros per year [2]. In addition, CVD and its sequelae place an additional economic burden on societies with non-health-care costs related to work absenteeism, presenteeism, premature mortality of 48 billion Euros [2]. To reduce the enormous clinical and economic burden of CVD, health services have increasingly focused on disease prevention in otherwise healthy individuals. As a first step, strategies mainly rely on assessment tools such as the Framingham score or the Systematic Coronary Risk Evaluation (SCORE) tool, which estimate a risk for future CVD events [3–5]. To reduce modifiable risk factors for people at higher risk, lifestyle changes can be recommended to inform clinical decision making for targeting preventive medication [6–8]. Studies, however, indicate potential inconsistencies between different risk estimation methods and guidelines [9–11]. Most predictive models were developed based on a combination of known risk factors and non-cardiac specific demographic determinants in large populations. The predictive accuracy in a population other than the development cohort can be challenging and limit a more universal applicability [12]. For the SCORE algorithm, which is one of the few tools that have been externally validated for CVD risk assessment, the ratio between observed and expected events varied between 0.28 and 1.50 in validation studies [12]. Indications for statin therapy can vary substantially based on various risk tools and may cause over- or underuse of medication in practice [10]. Lack of effectiveness data for different populations and regions, non-cardiac specific predictors, a residual risk that is not predicted by the classical determinants can hinder implementation into clinical practice or the development of screening programs [13, 14]. Although the SCORE algorithm, which is endorsed by the ESC, is the most widely established risk assessment in Europe [7, 12], several additional barriers to more optimal utilization have been identified such as financial barriers, lack of time and resources for primary prevention [13]. In addition, cost-effectiveness of preventive recommendations based on risk assessment lacks robust evidence suggesting a need for further economic evaluations of improved screening approaches [15].

Some of the barriers may be addressed by adding the information of a cardiac-specific biomarker [14]. Measurement of hsTnI has become available and hsTnI levels are detectable in over 90% of the general population [16]. Current studies have reported that hsTnI levels are associated with long-term CVD outcomes and suggested that high levels indicate a subclinical myocardial injury [16–21]. The Biomarker for Cardiovascular Risk Assessment in Europe (BiomarCaRE) project pooled ten population-based cohort studies and demonstrated that adding a person’s value of hsTnI to the SCORE prediction method led to improvements in discrimination (C-index) and net re-classification (NRI) for clinical endpoints [22]. This additive value of the biomarker was described as one of the eligibility requirements for CVD risk stratification [14]. Although C-index and NRI are useful measures to understand the incremental prognostic value of a biomarker [23, 24], a person will not directly benefit from improvements in a prognostic test unless it leads to changes in medical decision making and patient management [25]. Therefore, estimating the health impact of better testing requires more complex analyses, and outcome modelling and health-economic evaluation have been suggested as crucial tools for evaluation [26–29].

The objectives of this study were to perform a decision-analytic modelling study exploring potential changes in patient care and assessing the effect on health outcomes and the incremental cost-effectiveness of taking into account hsTnI in addition to SCORE for the prediction of CVD.

## Materials and methods

### Population

We included person-level information from asymptomatic individuals derived from European population-based cohort studies of the BiomarCaRE project as described elsewhere [22, 30]. In brief, the individual cohorts were the MONICA Brianza Study, the FINRISK Study, the DanMONICA Study, the Cooperative Health Research in the Augsburg Region (KORA) study, the Moli-sani Study, the Prospective Epidemiological Study of Myocardial Infarction from Belfast (PRIME), the Scottish Heart Health Extended Cohort Study (SHHEC), the MATISS (Malattie cardiovascolari ATerosclerotiche, Istituto Superiore di Sanità) Rome study, and the Northern Sweden MONICA Study. The baseline surveys were carried out between 1982 and 2010, and the follow-up information was collected in the most recent survey in 2011. Each cohort is based on a well-defined population, and their data were harmonized in the Monica Risk Genetics, Archiving and Monograph (MORGAM) Project [31]. Further details on the individual cohorts and the timing of baseline and follow-up examinations are provided in S1 Box in S1 File and summarized in S2 Table in S1 File. Serum troponin I was determined in the central BiomarCaRE core laboratory using a highly sensitive troponin I immunoassay (Abbott Laboratories, Abbott Park, Illinois, USA) [22]. The baseline dataset contained 87,808 observations (S2 and S3 Tables in S1 File). Only individuals with complete data required to compute SCORE, hsTnI, or CVD related outcome or death were included in the model cohort. Individuals with examination age below 20 years or above 85 years (N = 152), and individuals with CVD at baseline (N = 3,332) were excluded.

### Ethics statement

This study is based on a retrospective analysis of fully anonymized data from participating studies of the BiomarCaRE project. It complies with the Declaration of Helsinki that for all participating studies, the locally appointed ethics committee has approved the research protocol and that informed written consent has been obtained from the subjects (or their legally authorized representative). Data were compiled from the BiomarCaRE dataset on Feb 19^th^, 2019. More information is provided in S1 Box in S1 File.

### Principal model design

**Fig 1:**
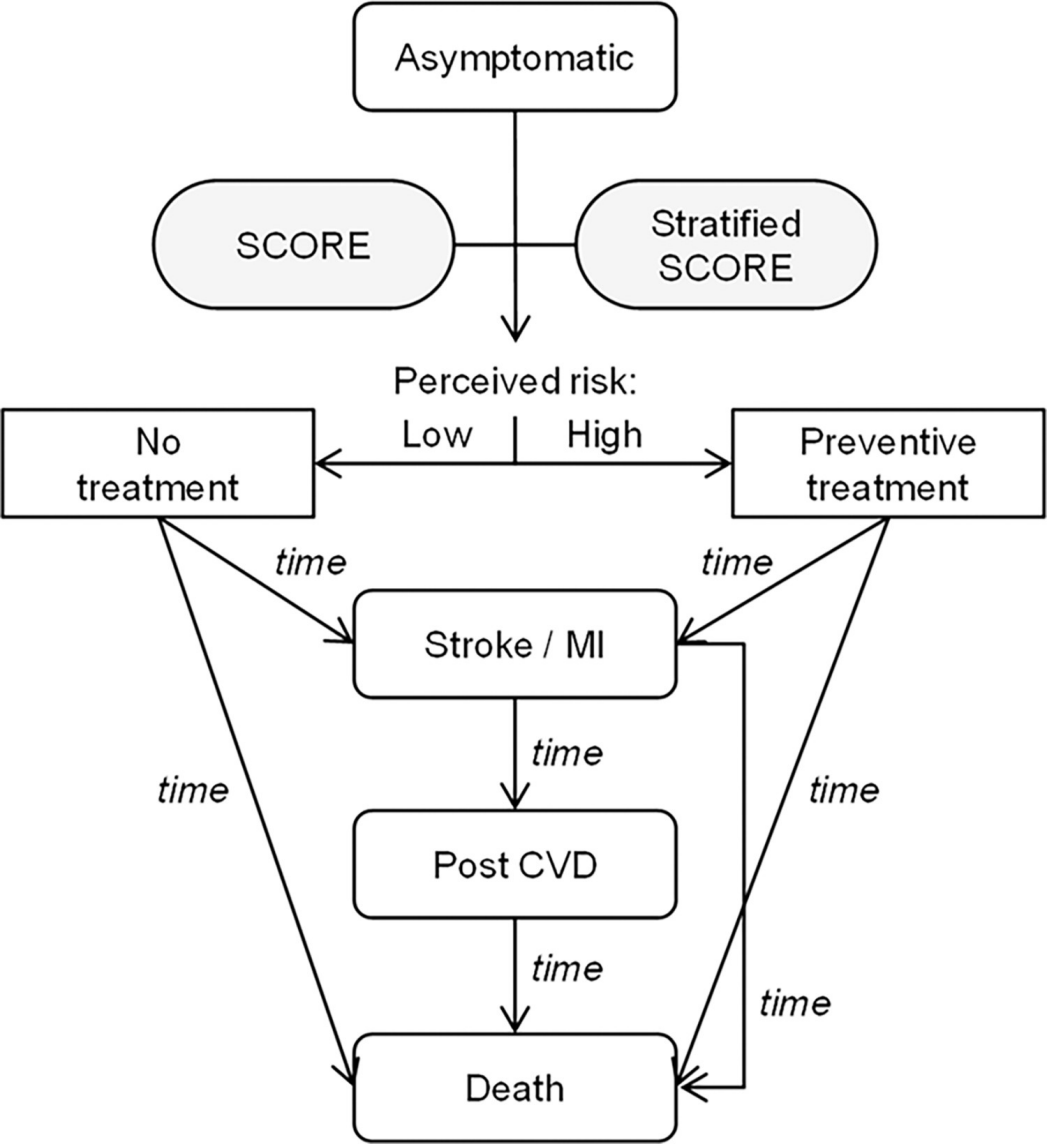
Principal model structure.

To consider the heterogeneity of individual characteristics in an asymptomatic population and competing time-dependent risks, we developed a de-novo discrete-event microsimulation model. The model compared two strategies for assessing the risk for cardiovascular events for asymptomatic, apparently healthy persons over a 10-year time horizon. The standard strategy reflecting current practice was employing the European risk assessment score (SCORE) [3]. According to the estimated risk, subjects were assigned to one of four SCORE risk classes (Low: <1%; Moderate (Mod): 1% to <5%; High: 5% to <10%; Very High: ≥10%) (SCORE in Table 1). The alternate strategy was adding hsTnI values to SCORE for further risk stratification (Stratified SCORE, S-SCORE), which was based on gender-specific high- and low-risk thresholds for hsTnI as suggested in a large population-based cohort study (Low risk: hsTnI <4 ng/L for women, < 6 ng/mL for men; moderate risk: 4–10 ng/L (women), 6–12 ng/L (men); high risk: > 10 ng/L (women), > 12 ng/L (men)) [19]. Stratification for baseline risk classes with hsTnI led to 11 risk classes in the alternative strategy (S-SCORE in Table 1). All strategies and assigned risk classes are summarized in Table 1. Individuals entered the model and were assessed for CVD risk with SCORE in the standard strategy or S-SCORE in the alternative strategy. As actual management and treatment information was not available from participants of the BiomarCaRE cohort, management assumptions in the standard strategy were based on guideline recommendations, that is, preventive drug treatment is recommended for subjects at very high risk, and should be considered for those in the high-risk class [7]. Accordingly, we assigned a probability for preventive medication of 50% and 100% to individuals with an estimated risk 5–10% (Risk class: High) and ≥10% (Very High), respectively. No intervention was considered for individuals with a risk below 5% (Low, Mod). In the S-SCORE strategy, probabilities for preventive treatment compared to the standard strategy were decreased with hsTnI values below the threshold (Low-, Mod-, High-), and increased in cases with elevated hsTnI (Low+, Low++, Mod+, Mod++, High+, High++). It was assumed that hsTnI values did not lead to changes in management in the highest risk class (Very High-, Very High+, Very High++) (Table 2). Following risk assessment, for each individual, the microsimulation tracked the occurrence of and the time to cardiovascular events (CHD, stroke) or death within an analytic time horizon of 10 years of follow-up. Strategies were compared in terms of a change in management, cumulative incidence of CVD events, CVD related mortality, and event-free years (per 1,000 persons). Reported effect measures include mean differences, relative risk reduction (RRR), and number needed to screen (NNS) to prevent one CVD event. In addition, the potential years of working life lost (PYWLL) were determined. The PYWLL refer to all years lost due to CVD related premature death before an assumed retirement age of 65 years. Quality-of-life index weights, so called utilities, were assigned to each state in order to derive 10-year quality-adjusted life years (QALY). Total direct medical costs for screening, preventive management, and treatment of events were calculated from a third-party payer’s perspective. Incremental cost-effectiveness ratios (ICER) were used to compare additional costs (€) divided by QALYs gained during the 10-year follow-up, comparing the S-SCORE with the SCORE strategy. A willingness-to-pay (WTP) threshold of 50,000 €/QALY was assumed [32]. Incremental Net Monetary Benefits (INMB) were calculated by multiplying the incremental QALYs with the WTP and subtracting the incremental costs. A positive INMB indicated that the alternative strategy S-SCORE was cost-effective compared to SCORE. The principal structure of the decision-analytic model is illustrated in Fig 1.

**Table 1:** Model comparators and risk classes.

Strategy	Risk class	SCORE risk	hsTnI value
SCORE	Low	<1%	n/a
S-SCORE	Low (-)	<1%	<4 (F), <6ng/L (M)
S-SCORE	Low (+)	<1%	4–10 (F), 6-12ng/L (M)
S-SCORE	Low (++)	<1%	>10 (F), >12ng/L (M)
SCORE	Mod	1 to <5%	n/a
S-SCORE	Mod (-)	1 to <5%	<4 (F), <6ng/L (M)
S-SCORE	Mod (+)	1 to <5%	4–10 (F), 6-12ng/L (M)
S-SCORE	Mod (++)	1 to <5%	>10 (F), >12ng/L (M)
SCORE	High	5 to <10%	n/a
S-SCORE	High (-)	5 to <10%	<4 (F), <6ng/L (M)
S-SCORE	High (+)	5 to <10%	4–10 (F), 6-12ng/L (M)
S-SCORE	High (++)	5 to <10%	>10 (F), >12ng/L (M)
SCORE	Very high	≥10%	n/a
S-SCORE	Very high (-)	≥10%	<4 (F), <6ng/L (M)
S-SCORE	Very high (+)	≥10%	≥4 (F), ≥6ng/L (M)

Baseline risk classes were derived from estimated risk of fatal CVD by using SCORE. In the alternative strategy S-SCORE, baseline risk classes were stratified by applying gender-specific, high, and low risk thresholds for hsTnI [17]. Mod: Moderate. F: Female, M: Male.

**Table 2:** Model input values and assumptions.

Variable	Base case	SA Range	Distribution		Reference
			1^st^ order	2^nd^ order	
Sex (% male)	48.7	±10%	Binomial	Beta	Model cohort
Time horizon, years	10		Fixed		
**Outcome probabilities (10Y)**					
CHD (Female/Male), %	2.4 / 6.6	Est. 95%CI	Weibull	Uniform	Model cohort (S4 Table in S1 File)
Stroke (Female/Male), %	0.8 / 1.4	Est. 95%CI	Weibull	Uniform	Model cohort (S4 Table in S1 File)
Death before CVD (Female / Male), %	3.6 / 5.9	Est. 95%CI	Weibull	Uniform	Model cohort (S4 Table in S1 File)
Fatal CHD events (Female/Male), %	32.7 / 28.0	±10%	Binomial	Normal	Model cohort
Fatal stroke events (Female/Male), %	10.8 / 10.3	±10%	Binomial	Normal	Model cohort
**Post-event mortality, %**					
CHD: Age < 55, Female/Male,	11.6 / 9.4	±10%	Weibull	Normal	[33]
CHD: Age 55–74, Female/Male	30.0 / 29.0	±10%	Weibull	Normal	[33]
CHD: Age 75+, Female/Male	60.9 / 63.2	±10%	Weibull	Normal	[33]
Stroke: Age < 55, Female/Male	12.0 / 14.7	±10%	Weibull	Normal	[33]
Stroke: Age 55–74, Female/Male	35.3 / 37.6	±10%	Weibull	Normal	[33]
Stroke: Age 75+, Female/Male	61.1 / 68.2	±10%	Weibull	Normal	[33]
**Preventive treatment probability, %**					
SCORE risk class: Low	0		Fixed	Fixed	[7]
S-SCORE risk class: Low-	0		Fixed	Fixed	Assumption
S-SCORE risk class: Low+	1	1–25	Binomial	Beta	Assumption
S-SCORE risk class: Low++	30	1–50	Binomial	Beta	Assumption
SCORE risk class: Mod	0		Fixed	Fixed	[7]
S-SCORE risk class: Mod-	0		Fixed	Fixed	Assumption
S-SCORE risk class Mod+	75	50–99	Binomial	Beta	Assumption
S-SCORE risk class Mod++	99	50–99	Binomial	Beta	Assumption
SCORE risk class: High	50	30–75	Binomial	Beta	[7]
S-SCORE risk class: High-	30	1–50	Binomial	Beta	Assumption
S-SCORE risk class: High+	75	50–99	Binomial	Beta	Assumption
S-SCORE risk class: High++	99	75–99	Binomial	Beta	Assumption
SCORE risk class: Very high	100		Fixed	Fixed	[7]
S-SCORE risk class: Very high-	99	75–99	Binomial	Beta	Assumption
S-SCORE risk class: Very High+ & Very High++	100		Fixed	Fixed	Assumption
Risk ratio of preventive treatment	0.65	0.58–0.73	LogNormal	Beta	[34]
**Utilities**					
Asymptomatic	0.96		Beta	Fixed	Assumption
Acute CHD decrement	0.15	0.13–0.17	Beta	Beta	[35]
Acute stroke decrement	0.19	0.16–0.22	Beta	Beta	[35]
Post-CHD	0.82	0.78–0.86	Beta	Beta	[35]
Post-stroke	0.52	0.44–0.60	Beta	Beta	[35]
Preventive drug treatment annual decrement	0.01	0.008–0.012	Beta	Beta	[36]
**Costs, 2019€**					
One-time health check and screening	44	39–48	Fixed	Normal	[37]
Incremental for utilizing hsTnI	25	10–50	Fixed	Normal	Assumption
Preventive treatment per year	595	536–655	Fixed	Normal	[38]
CHD (first year)	15,805	15,278–16,332	Gamma	Normal	[39]
CHD (2^nd^ year)	2,318	±25%	Gamma	Normal	[40]
CHD (Years 2+)	600	±25%	Gamma	Normal	[40]
Stroke (1^st^ year)	21,724	19,210–24,239	Gamma	Normal	[39]
Stroke (2^nd^ year)	13,528	±25%	Gamma	Normal	[39, 40]
Stroke (Years 2+)	8,571	±25%	Gamma	Normal	[39]
Annual discount rate, %	3	0–5	Fixed		

SA: sensitivity analysis. All costs in 2019 €. Estimated Weibull parameters for time-to-event functions are provided in S4 Table in S1 File. Model cohort: BiomarCaRE subgroup included in the study.

### Time-to-event

Event times for all subjects included in the model cohort were analyzed with the Kaplan-Meier method stratified by gender and type of event (stroke, CHD, death before CVD), based on the individual data. Parametric Weibull survival functions were also estimated. For Weibull parametrization, a follow-up of 15 years was considered. Event rates for the 10-year time horizon computed from the Weibull parametrization were compared to those using the respective Kaplan-Meier estimator (S4 Table in S1 File). In the model, event rates were individually sampled from the parametric Weibull functions. This approach was assumed to reflect the standard risk of the SCORE strategy in the model and was validated against observed event rates in the study cohort (S5 Table in S1 File). More details are provided in the section about the Sampling Strategy and in S1 Fig in S1 File.

### Fatality of events and post-event mortality

The proportions of fatal CHD and stroke events were evaluated from the model cohort and were applied in case an event occurred by sampling from respective beta-distributions. Event type (CHD, stroke), sex, and age-group specific post-event mortalities were retrieved from a Dutch cohort study that was evaluating the dynamics of mortality of myocardial infarction and stroke over follow-up time [33]. Data were transformed into a Weibull parametrization using the cumulative density function $F(t)=1-e^{-\lambda t^{k}}$ with the shape parameter k fitted to reported 1-year and 5-year data stated in the study. Scale parameters *λ* were calculated accordingly. Individual post-event times were retrieved by sampling from the resulting Weibull distribution.

### Treatment effectiveness

The impact of preventive drug treatment in the S-SCORE strategy was considered by applying a hazard ratio to time-to-event functions for CHD and stroke for individuals who were managed differently in S-SCORE compared to the standard SCORE. A reduction in risk was assumed for individuals not treated in the SCORE strategy but assigned to treatment in S-SCORE. An increase in risk was applied to those treated in SCORE but not assigned to treatment in the alternate strategy S-SCORE (S2 Box in S1 File). Risk ratios (RR) were taken from a Cochrane systematic review of statins for primary prevention of CVD [34], and were sampled from a log-normal distribution for each subject in the study. In case of changed management, the impact on time-to-event was calculated from the risk ratio of statin treatment and the Weibull cumulative density distribution F(t) as follows: RR = p’(Event) / p(Event) = F’(10Y) / F(10Y), where F and F’ denote the cumulative probabilities of CVD after 10 years of follow-up of the standard and alternative strategy, respectively. With $F(t)=1-e^{-\lambda t^{k}}$, where λ and k represent the Weibull scale and shape, and assuming a constant k, time-to-event function scale parameters were modified in the alternate strategy S-SCORE with RR = λ’ / λ.

### Health state utilities

Health state utilities (HSUs) are required for calculating quality-adjusted life years. Although HSU for cardiovascular events are available in the published literature, values vary substantially due to variation in definitions of clinical events, settings, elicitation tools, and countries. For our purpose, health state utilities were informed by a study in which UK general population respondents valued acute and chronic cardiovascular health states in time trade-off tasks [35]. In this study, an acute state was described by an event plus subsequent treatment and recovery in the first year. A chronic state was referred to as stable health with a duration of 10 years after an acute event. The cumulated utilities over 10 years were linearly back calculated to time zero for estimating absolute utility decrements for acute CHD and stroke events. Time dependent values were taken from chronic state HSUs. Utility decrements were also applied to individuals on preventive drug treatment [36]. Individual utility weights were assigned by sampling from beta distribution. 10-year QALYs were derived from the model based on survival and HSUs and were discounted at a constant rate of 3% per annum [41].

### Direct medical costs

The evaluation of costs was performed form a third-party payer’s perspective, and therefore, considered direct medical costs only. Costs for preventive treatment [38], health check and screening [37] were derived from independent studies for Germany. Treatment and post-event costs for CHD and stroke were divided into acute phase costs, costs in the first year, second year, and years following the second, and were informed by a systematic review [39] and additional evaluations [40, 42, 43]. All costs were adjusted to 2019 Euro assuming a 2% inflation rate. Future costs were discounted at 3% per annum. Costs were randomly sampled for each individual from respective distributions assuming a coefficient of variation of 10%.

### Model calculation and validation

The estimation of survival functions (Kaplan-Meier and Weibull) was conducted using R software version 3.5.1. [44]. The decision-analytic model was developed in TreeAge Pro 2018 (TreeAge Software, Williamstown, MA, USA). Further statistical analyses were performed in Minitab 17.1.0. (Minitap, Ltd. Coventry, UK). In order to assess the model’s accuracy for making relevant and meaningful predictions, the decision-analytic model was validated in several steps [45]. Experts (ACF, US) were reviewing model structure, input assumptions, data sources, formulas, and results. Model structure was informed by an extensive literature review [46]. Individual tracker variables were used to capture modeled individual outcomes and to validate model calculations. The model was validated by comparing simulated results for the standard strategy (SCORE) with cumulative incidences of CVD events and death as observed in the underlying cohort retrieved from the BiomarCaRE dataset.

### Sampling strategy, sensitivity analyses and subgroup analyses

The decision analysis was performed using 1^st^-order Monte Carlo microsimulation. Stratified by gender, event times were randomly sampled per each trial from competing time-to-event distributions for CHD, stroke, and death. According to the earliest event within a ten-year time horizon, respective individuals were bootstrapped from the model cohort with their baseline characteristics (age, estimated risk from SCORE, hsTnI value). Model assumptions were sampled individually from 1^st^ order distributions as stated in Table 2. In the base-case analysis, expected values were calculated from a series of 100 independent runs, each consisting of 20,000 individuals. This approach was used to obtain statistically robust results while considering a maximum of information available from the model cohort, and a manageable modelling time. An illustration of the sampling strategy of the base-case analysis is shown in S1 Fig in S1 File. Probabilistic sensitivity analysis (PSA) was performed using 2^nd^ order Monte Carlo simulation and included 500 iterations of a microsimulation with 20,000 individuals each, but in contrast to the base-case analysis, second-order uncertainty was considered by varying input assumptions in each run by randomly retrieving values from the respective 2^nd^-order distributions (Table 2), assuming independency of variables. The base-case analysis did not provide individual data, so we collected individual attributes from a microsimulation of 250,000 samples and performed subgroup-analyses for different age and risk groups (S10 Table in S1 File).

In addition, we analyzed a scenario not discounting for costs and QALYs and a scenario with an extended analytic time horizon of 15 years. A Derived Management scenario (DM) was evaluated by using preventive treatment probabilities that led to the most preferable INMB in univariate SA (S19 Table in S1 File). Comparisons between strategies were made based on the respective means and 95% confidence intervals. Statistical significance was analyzed conducting a 2-sample t-test with a significance level of 0.05. Confidence intervals were calculated or estimated from the 2.5^th^ and 97.5^th^ percentile of result distributions derived from the 2^nd^-order Monte Carlo simulation. The probability for the compared strategies being cost-effective were assessed within a WTP range from 0 to 100,000€ per QALY gained and presented as cost-effectiveness acceptability curves (CEAC). Sensitivity analyses were conducted to consider parameter variability and to check the impact of individual variable uncertainty on results and the overall model robustness. Univariate sensitivity analyses were performed on all variables by varying input values in independent microsimulation runs with 100,000 samples between the upper and lower bound of the model input parameters as stated in Table 2. Results of univariate sensitivity analyses (SA) were reported as INMB tornado diagrams relative to the lower bound. In addition, full base-case sampling approach (100 runs, 20,000 bootstrapped samples each) were applied to varying input values of parameters regarded as relevant. We followed the ISPOR-SMDM Task Force guidelines on good research practices in decision-analytic modeling [47, 48] and the Consolidated Health Economic Evaluation Reporting Standards (CHEERS) for the reporting of study results [49].

## Results

### Model validation and base-case analysis

From the BiomarCaRE dataset, 72,190 individuals were included in the model cohort with baseline characteristics described in Table 3. The cohort consisted of 48.7% men at an average age at baseline of 50.6 years. Over a follow-up of ten years, a CVD event occurred in 5.4% of the individuals. For validation, the model output demonstrated an excellent concordance to the observed number of CVD events, CHD events, strokes, and deaths (S5 Table in S1 File). Differences in management and outcome between the S-SCORE strategy and the standard strategy SCORE are shown in Table 4. In SCORE, 9.4% of subjects received preventive medication, whereas 14.5% were assigned to medication in the alternate strategy S-SCORE. A change in management was indicated for a total of 10.0% of subjects. While 7.5% of people not treated in SCORE were assigned to higher risk and received preventive medication in S-SCORE, 2.5% of subjects treated in SCORE had their risk category downgraded to become ineligible for treatment in S-SCORE. More information regarding management is presented in S6 and S7 Tables in S1 File. The cumulative incidence of CVD events was reduced from 5.38% in SCORE, compared to 4.85% in S-SCORE (p<0.001). A statistically significant reduction was observed for all clinical endpoints for CHD events (3.90% vs. 4.36%, 95%CI of difference: -0.49% to -0.41%), stroke (0.94% vs. 1.02%, CI of difference: -0.10% to -0.06%), CVD related mortality (2.14% vs. 2.38%, CI of difference: -0.27% to -0.21%), and all-cause mortality (6.80% vs. 7.04%, CI of difference: -0.29% to -0.19%). In addition, S-SCORE led to a gain of 23 (95%CI: 20 to 26) event-free years per 1,000 persons screened in 10 years of follow-up. Over the same period, S-SCORE reduced the potential years of working life lost (PYWLL) due to premature death by 4.5% and saved 5 years (95CI: 3 to 8) per 1,000 persons. In summary, the S-SCORE strategy led to a relative risk reduction (RRR) for CVD events of 9.9% (95%CI: 7.3 to 13.5%). The number needed to screen (NNS) to prevent one CVD event with S-SCORE compared to SCORE in 10 years follow-up was 183 (95%CI: 172 to 203). For evaluating overall health outcomes, S-SCORE gained 7 (95%CI: 5 to 9) QALYs per 1,000 subjects screened. The S-SCORE strategy increased the average costs per subject by 187 € (95%CI: 177 € to 196 €), when compared to the standard SCORE. In total, this led to an ICER in the discounted base-case analysis of 27,440 € per QALY gained which is below the assumed WTP threshold of 50,000 € (Table 5).

**Table 3:** Baseline characteristics of the model cohort. If not otherwise stated, continuous variables are presented as median, 25th percentile, and 75th percentile. Binary variables are described as absolute and relative frequencies.

	All (N = 72,190)	Men (N = 35,173)	Women (N = 37,017)
Examination age (years)	50.6 (41.3, 59.2)	50.9 (41.7, 59.2)	50.1 (41.0, 59.2)
Survey year, range	1982–2010	1982–2010	1982–2010
Male No. (%)	35,173 (48.7)	35,173 (100)	0 (0)
BMI (kg/m^2^)	26.2 (23.5, 29.3)	26.5 (24.3, 29.1)	25.7 (22.8, 29.5)
Systolic BP (mmHg)	131.0 (119.0, 146.0)	133.5 (122.0, 147.0)	128.0 (116.0, 145.0)
Total cholesterol (mmol/L)	5.7 (5.0, 6.5)	5.7 (5.0, 6.5)	5.7 (5.0, 6.5)
Diabetes No. (%)	2,792 (3.9)	1,502 (4.3)	1,290 (3.5)
Daily smoker No. (%)	19,046 (26.4)	10,088 (28.7)	8,958 (24.2)

**Table 4:** Management and CVD related outcomes.

Outcome	SCORE	S-SCORE	Difference	
			Mean	95%CI
Preventive treatment, %	9.40	14.46	5.05	(4.98; 5.12)
Changed management vs. SCORE, %			10.02 (Off treatment: 2.5; To treatment: 7.5)	(9.98; 10.07)
CVD, %	5.38	4.84	-0.54	(-0.58; -0.49)
CHD, %	4.36	3.90	-0.45	(-0.49; -0.41)
Stroke, %	1.02	0.94	-0.08	(-0.10; -0.06)
All-cause mortality, %	7.04	6.80	-0.24	(-0.29; -0.19)
CVD related mortality, %	2.38	2.14	-0.24	(-0.27; -0.21)
EFS, years p. 1,000 pers.	9,572	9,595	23	(20; 26)
PYWLL per 1,000 pers.	115	110	-5	(-8; -3)

EFS: Years of event free survival in 10 years of follow-up. PYWLL: Potential years of working life lost due to premature death assuming a retirement age of 65. All differences p<0.001. More details on management changes between strategies are given in S6 and S7 Tables in S1 File.

**Table 5:** Cost-effectiveness results. The 95% confidence intervals for the ICER were estimated from 2.5th and 97.5th percentiles.

Strategy	Costs		QALY (per 1,000 persons)		Incr. Costs (per person)		Incr. QALYs (per 1,000 persons)		ICER	
	Mean	95%CI	Mean	95%CI	Mean	95%CI	Mean	95%CI	Mean	95%CI
SCORE	1,349	(1342; 1356)	8,214	(8213; 8216)						
S-SCORE	1,536	(1529; 1543)	8,221	(8220; 8223)	187	(177; 196)	7	(5; 9)	27,440	(13,429; 123;027)

ICER: incremental cost-effectiveness ratio

### Sensitivity analyses

**Fig 2:**
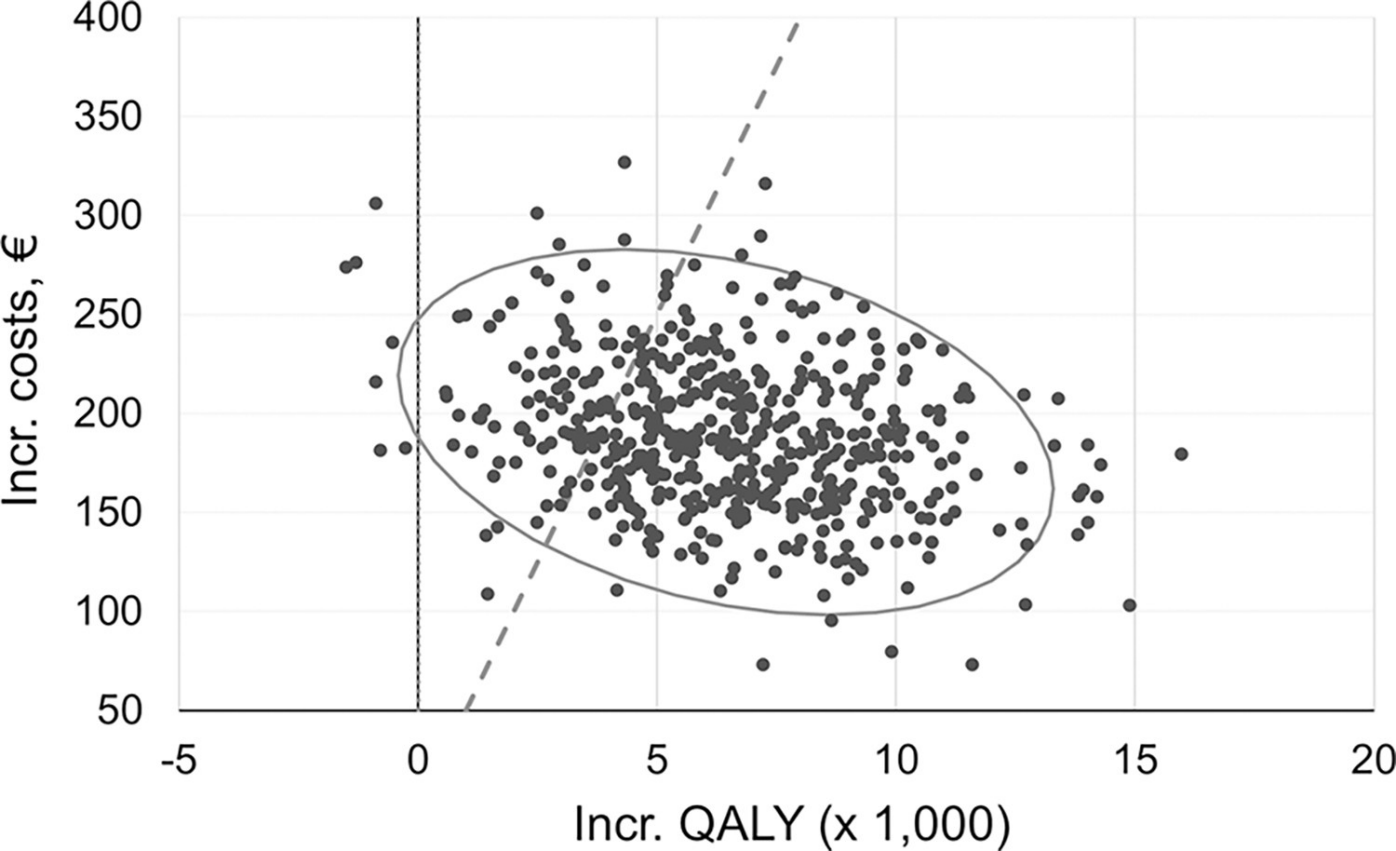
Incremental cost-effectiveness scatter plot from probabilistic sensitivity analyses. Probabilistic sensitivity analysis (PSA) with 500 iterations of microsimulations with 20,000 samples. Each dot is a single iteration. Dashed line represents the willingness-to pay threshold of 50,000 € per QALY. Incr.: Incremental.

**Fig 3:**
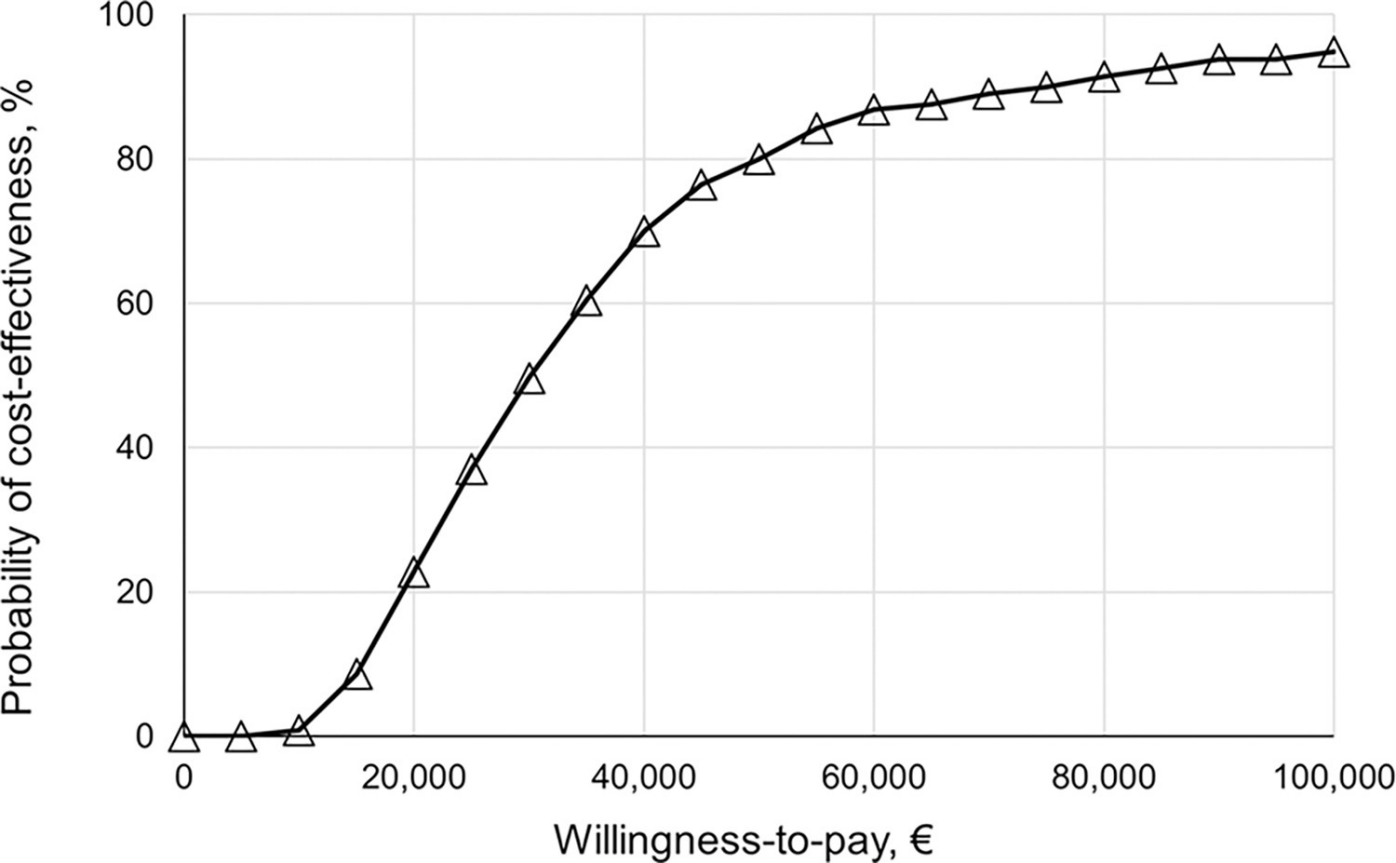
Cost-effectiveness acceptability curve of the alternative strategy S-SCORE. At a willingness-to-pay threshold (WTP) of 50,000 € per QALY gained, the S-SCORE strategy had a probability of 80% of being cost-effective.

**Fig 4:**
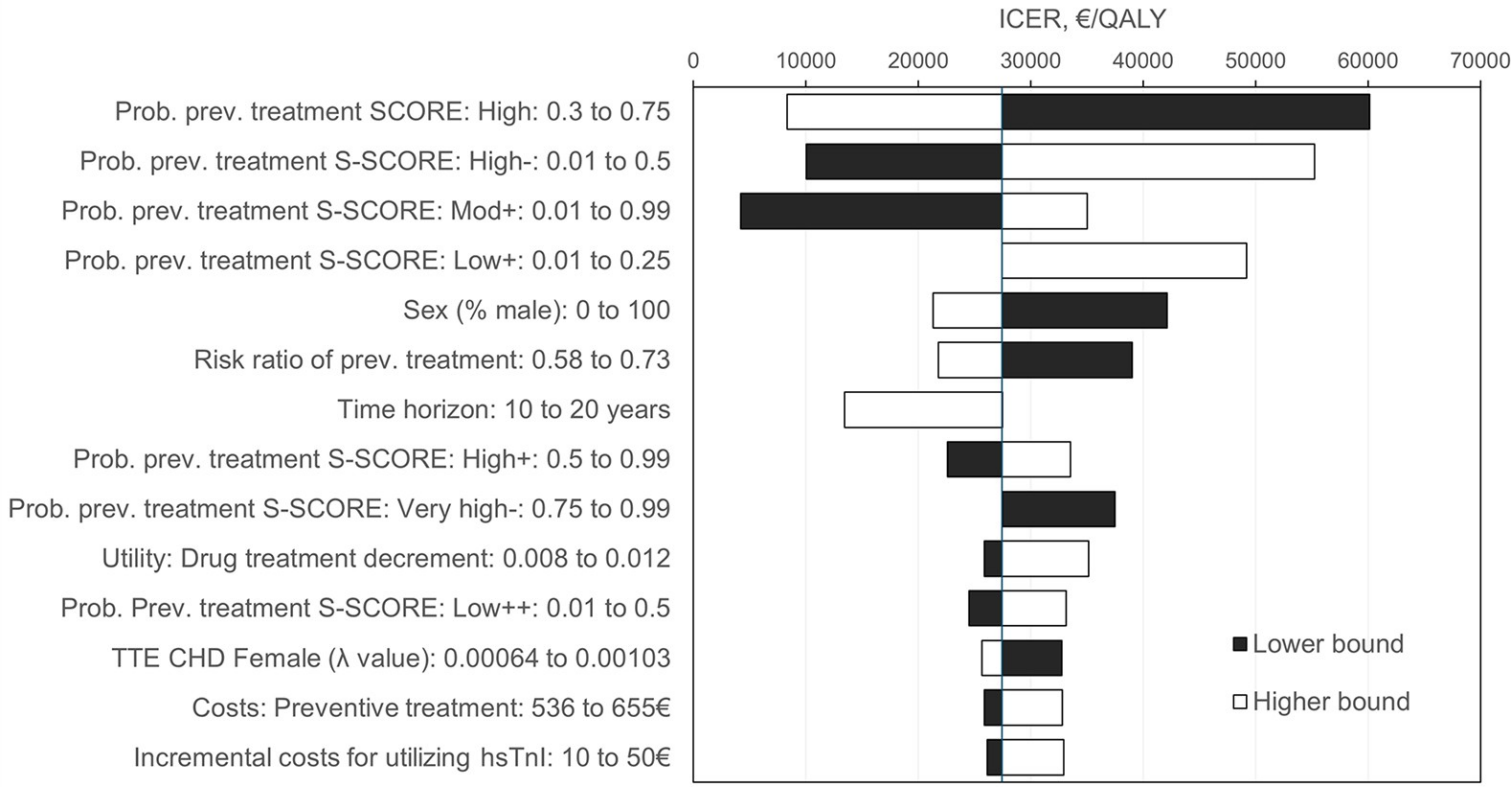
Sensitivity analyses tornado diagram assessing the influence of variables on the incremental cost-effectiveness ratio. Independent analyses (100 repeated microsimulation runs with 20,000 bootstrapped samples) for each parameter value varied between the lower and higher bound. Incremental cost-effectiveness ratio (ICER) for S-SCORE vs. SCORE. The base-case analysis is indicated by the vertical line at 27,440 € per QALY gained. The diagram shows variables with a difference in ICER compared to the base-case analysis of at least 10% in any direction.

Probabilistic sensitivity analysis (PSA) confirmed the significant changes in management and clinical endpoints by S-SCORE (Fig 2). Detailed PSA results are shown in S8 Table in S1 File. At a WTP of 50,000 € per QALY gained, the S-SCORE strategy had a probability of 80% of being cost-effective (Fig 3). In 18.6%, the S-SCORE gained more benefits, but incremental costs were above the WTP threshold (S9 Table in S1 File). As demonstrated by univariate sensitivity analyses, model results were most sensitive to the probabilities of preventive medication in different risk classes, and the efficacy of treatment on preventing CVD events (Fig 4). A change in the preference from S-SCORE to SCORE was indicated for average treatment probabilities in the SCORE high-risk class below 35%, in the S-SCORE “High-” class above 48%, and in the S-SCORE “Low+” class at around 25% (Fig 4). Variation of input assumptions within the stated ranges for all other variables did not result in a preference change for S-SCORE. S4 Fig in S1 File shows the direction of influence when varying input parameters. With an increasing input value of the respective variable, a negative INMB indicates that S-SCORE is less preferred, while a positive INMB indicates a favorable effect of S-SCORE.

### Subgroup and scenario analyses

Reductions in CVD events were statistically significant for subjects aged 40 to 70 and at moderate or high SCORE risk (S14 Table in S1 File). The relative risk reduction (RRR) of S-SCORE vs. SCORE was higher for female than for male (12.1% vs. 8.7%), and it was highest in the subgroup of subjects aged 40 to 70 and in those at high risk (S19 Table in S1 File). The average total costs per person in S-SCORE compared to SCORE was lower for male between 61 and 80 years (S16 Table in S1 File) and for people at high SCORE risk and a hsTnI value below the lower thresholds (classified as High-; S17 Table in S1 File). Incremental QALYs of S-SCORE were highest for people at moderate SCORE risk and hsTnI > 10 or 12 ng/L (Mod++), and for male at high risk and hsTnI between 6 and 12 ng/mL (High+; S17 Table in S1 File). In people aged 40 to 70 at high SCORE risk and in those at high SCORE risk, S-SCORE dominated SCORE by gaining more QALYs at substantially lower costs (S17 Table in S1 File).

**Fig 5:**
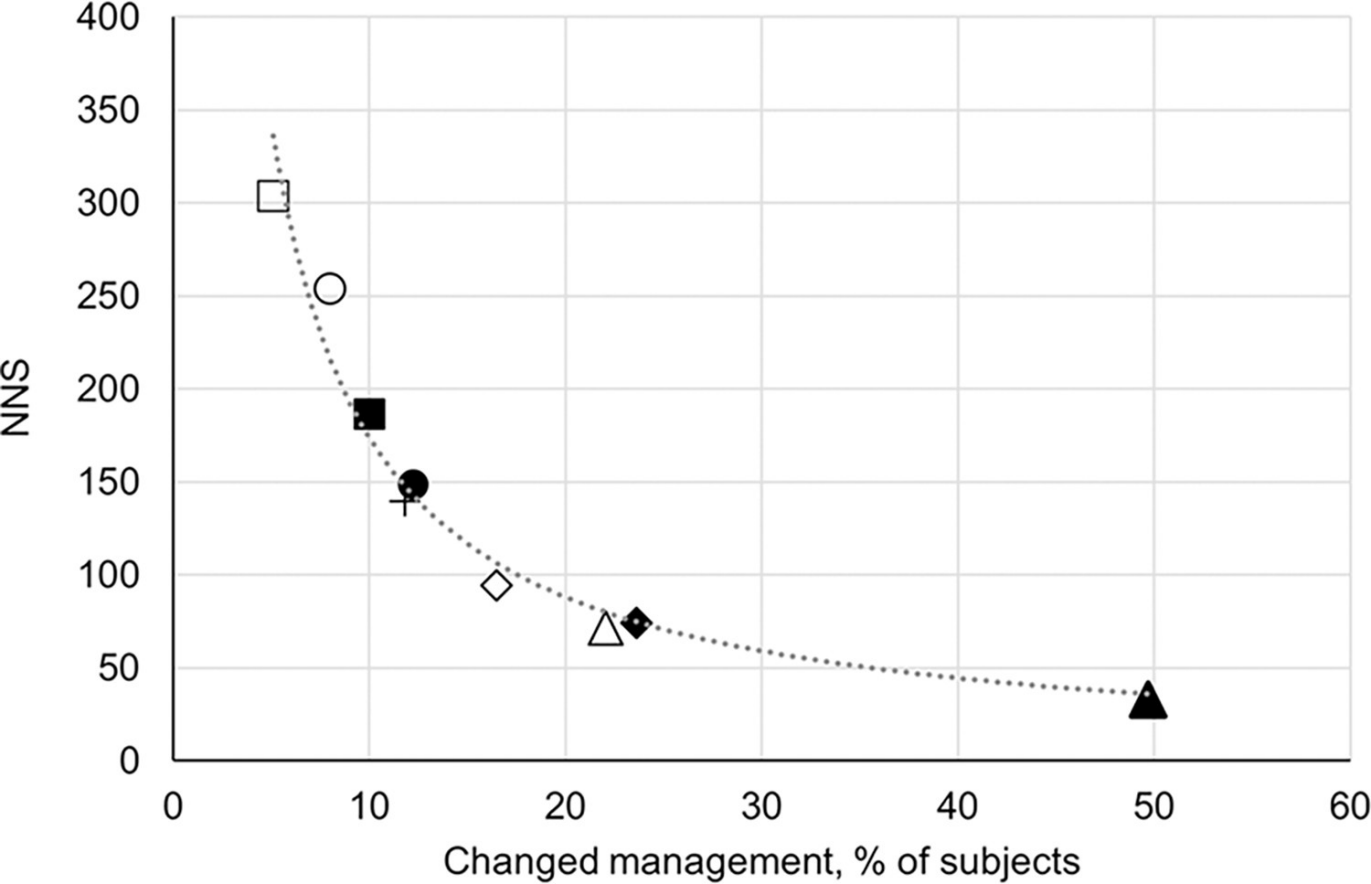
Proportion of subjects with a change in management in S-SCORE compared to SCORE and resulting number needed to screen. Base-case (◼); Female* (○); Male* (●); Age 40–70* (+); Moderate risk* (◊); Moderate & high risk* (◆); Age 40–70, moderate and high risk* (Δ); Age 40–70, high risk* (▲); Derived Management scenario (□). All analyses followed the base-case sampling approach except *, in which subgroup analyses were derived from a microsimulation including 250,000 individuals. NNS: Number needed to screen to prevent one event.

Extending the time horizon of the base-case analysis to 15 years, S-SCORE gained significantly more benefits (16.1 vs. 6.8 incremental QALYs per 1,000 screened subjects), was more costly (273 € vs. 187 € per screened subject), and with an ICER of 16,992 €/QALY it demonstrated substantially improved cost-effectiveness compared to the base-case analysis (S19 Table in S1 File). In the Derived Management scenario (DM), S-SCORE dominated SCORE by gaining more QALYs at lower costs compared to SCORE (S19 Table in S1 File). The proportion of subjects who were managed differently in S-SCORE compared to SCORE was clearly associated with the clinical outcome expressed as the number needed to screen to prevent one CVD event (NNS) (Fig 5): The higher the proportion of subjects with a change in management between both strategies, the lower the NSS.

## Discussion

In this decision-analytic study based on individual level data, we applied hsTnI in addition to SCORE for estimating a person’s risk of future CVD events (S-SCORE strategy), explored the potential changes in primary prevention management, and assessed how these affect health outcomes and cost-effectiveness compared to a strategy based on SCORE alone. We developed an individual-level discrete-event simulation populated with and validated against data from apparently healthy subjects of a large pan-European cohort (BiomarCaRE, [22, 30]). We showed that S-SCORE would lead to different clinical decisions for 10.0% of subjects, of which 75% were assigned to preventive treatment after being upgraded to a higher risk class. Resulting from changed management, all clinical endpoints considered in the study were significantly better for individuals screened with S-SCORE when compared to those screened with SCORE. Optimizing preventive actions for people at higher risk led to a reduction in risk for CVD events of 10% in 10 years following screening with a number needed to screen to prevent one event of less than 200. Linking reduced event rates to QALYs and considering costs, S-SCORE demonstrated a higher probability of cost-effectiveness relative to the standard strategy. These findings were consistent across a wide range in input assumptions, including assumptions about preventive management, and corroborated by extensive univariate and probabilistic sensitivity analysis.

Health economic models for primary prevention programs in asymptomatic people are challenged by a substantial heterogeneity in the population, low to moderate incidence or eligibility rates, common variability and uncertainty in management decisions, treatment effects, or course and severity of events. In our base-case analysis, all these effects were considered by a combination of bootstrapping individual characteristics from a very large cohort and assigning randomly sampled parameter values from respective distributions to each subject, offering insights, from the distribution of individual results, on the cost- effectiveness for subgroups of the model cohort and the robustness of our conclusions. This individual-level process was repeated numerous times. Thus, after re-iterating this procedure for generating several subsets, the final outputs reflected the outcomes for the entire population of interest. In our model, the base-case analysis consisted of 2 million individual samples which were analyzed in 100 independent model runs of 20,000 subjects each bootstrapped from the model cohort of 72,190 individuals. Validated against observed outcomes, and corroborated by probabilistic sensitivity analysis, this approach reflects heterogeneity and variability, and supports robust conclusions.

While recommended by guidelines [6, 7], the general evidence for CVD risk assessment programs appears to be inconclusive [12, 50–54]. This might be explained by some considerable variation in populations and study characteristics such as predicted outcomes, time horizons, included predictors, and reported performance measures. Some bias might also be caused by inaccuracy in the prediction that led to inappropriate management and medication [9, 53]. Adding a biomarker has been suggested to potentially address issues with risk stratification beyond established risk factors particularly for the elderly. To date, clinical trials that evaluate the potential benefits of adding hsTnI to existing risk assessment methods have not been conducted. It should be noted that the assessment of primary prevention programs would generally require long follow-up time. This and the aforementioned level of uncertainty makes health economic modelling a tool of choice [28, 47], and several studies have evaluated the cost-effectiveness of risk assessment tools [15, 55–57]. Against the fact that any modification to a program may impact cost, effectiveness, and cost-effectiveness, it is not surprising that a systematic review failed to aggregate information from cost-effectiveness studies for CVD risk assessment programs [15]. While most studies suggested cost-effective alternatives, the authors found a wide variety in terms of population, screening strategies and interventions, and they stressed the urgent need for more health economic evaluations. The vast majority of studies focused on aspects of implementation compared to no assessment [15, 55–57]. Only very few evaluations elaborated on the additional use of a biomarker besides the common consideration of cholesterol as a variable in risk assessment scores. One study described a test-and-treatment strategy that was targeting an asymptomatic middle-aged population and was guided by low LDL and elevated high-sensitivity C-reactive protein (hsCRP) [58]. Compared to a no-test-no-treat practice, this strategy gained 310 QALYs following 1000 subjects over a lifetime and was found cost-effective with an ICER of $25,198 per QALY (2009 US Dollar). While these results are in a range comparable to our findings, it should be noted that this study was suggesting an alternate strategy versus no testing and not against an established assessment method. In a recent cost-effectiveness study, a strategy was evaluated in which subjects were risk-stratified with hsTnI only and those at high-risk were sent to preventive medication [59]. Compared to a do-nothing strategy, this was found highly cost-effective and cost-saving in a country classified as low-risk and very high-risk for CVD, respectively. In both settings, the NNS to prevent one event ranged between 185 and 217 persons. Another study discussed the use of a hypothetical test to risk stratify subjects at SCORE risk from 3–15% [57]. The authors found an improvement in QALYs and by considering costs, they concluded that a prognostic test would have the potential to be more cost-effective than other strategies including the one proposed by the guideline. In summary and to our knowledge, ours is the first study that elaborates on the additional use of hsTnI in a risk assessment tool by estimating the impact on clinical decision making and the resulting level of cost-effectiveness based on a large and comprehensive cohort with individual-level data.

Assessment tools such as SCORE, which estimates the 10-year risk of fatal CVD risk [7], mainly rely on several non-specific risk factors such as lipids, blood pressure or age. Conclusions are derived from associations observed in large population studies. When applying these tools to other populations, several studies reported a degree of inaccuracy and poor calibration, under- or overestimating of risk, or inappropriate drug use [60, 61]. In most previous cost-effectiveness modeling studies, however, probabilities for CVD events were directly calculated from risk equations such as SCORE. Albeit relatively simple, this approach appears questionable in light of the uncertainty related to actual event probabilities. By contrast, the occurrence of events in our study was derived from observed outcomes. The estimated SCORE risks were only used for assigning subjects to respective risk categories. We believe that this distinct feature of our model mitigated issues related to inaccuracies in predicting an event, and therefore considerably increased the validity of findings.

In addition to general non-specific variables, the use of hsTnI, a heart specific marker for structural and functional alterations, further improved the risk stratification for future CVD events, and may therefore overcome some of the limitations and concerns [16]. Different cardiac troponin isoforms exist, but early studies indicate that high-sensitivity troponin I and troponin T have different associations with cardiovascular disease outcomes in the general population [62, 63]. An overview is given by Leite et al. who suggest that hsTnI seems to be a potential strong marker to complement cardiovascular risk charts [64]. As our study is an extension of the BiomarCaRE project [22], our study only considered the use of hsTnI.

It is important to note that the assumption that better risk prediction will improve outcomes is not a given. Whether altered risk assessment is clinically relevant, primarily depends on its impact on clinical decisions, which in fact would require a formal impact analysis [65]. Lacking robust data, most cost-effectiveness studies assumed that all eligible patients received preventive treatment. This is despite the fact that clinical guidelines are not strictly followed and indeed recommend an increase in the intensity of advice with increasing risk while also considering factors such as age [7]. A study evaluating the NHS health check program demonstrated that an optimal recommendation-based prescription scenario led to superior cost-effectiveness compared to what was usual care [66, 67]. This is in line with our findings that the probability of receiving preventive medication in eligible risk classes is a key driver for cost-effectiveness (Fig 4). In our study, we considered decision variability by applying an individual-level sampling approach. In addition, by comparing the base-case analysis with the scenario with the derived treatment probabilities, the effect of decision making became apparent and moved S-SCORE from being a cost-effective alternative to a cost-saving alternative (S19 Table in S1 File). Thus, our study explicitly describes the impact of a biomarker on decision making as an essential step to better CVD prevention, and results may be useful for exploring a more detailed study design for a prospective impact analysis. The step from information to decision deserves specific attention, and a better understanding of where a change in decision making occurs is an important aspect aiming for the most valuable approach.

As per our assumption in the moderate risk category, no subject received preventive medication in SCORE, and all subjects whose risk category was upgraded in S-SCORE were referred to treatment (16.5% in S7 Table in S1 File). In conjunction with clinical outcome data (S14 and S19 Tables in S1 File), this indicates that SCORE underestimated the actual risk in a number of cases at moderate risk. Consequently, in subjects with a moderate SCORE risk, the high-risk threshold for hsTnI (>10ng/mL for female, >12ng/mL for male) clearly identified those who benefited most from S-SCORE (S19 Table in S1 File). In the high-risk category, half of the people were managed differently with S-SCORE compared to SCORE, of whom 58% were withdrawn from preventive medication, which suggests an overutilization of drugs in the SCORE risk group between 5–10%. The relationship between test information, decision-making and outcome is revealed by comparing different subgroups and scenarios (Fig 5). We observed a clear association between a change in management caused by hsTnI in S-SCORE and the clinical outcome expressed as NNS. While this underscores that the management assumptions in the model were appropriate, it also confirms that decisions guided by hsTnI can effectively change the outcome. In addition, it emphasizes the importance of identifying the optimal management scheme.

The European SCORE was fitted to the middle-age group 45–64 years [3], and guidelines recommend risk assessment for men and women with no known risk factors aged >40 and >50, respectively [7]. Also, the assessment of troponin I for CVD risk prediction was not regarded useful below the age of 45 [22]. Studies have confirmed a positive effect of targeted approaches on cost-effectiveness [37]. In our study, all statistically significant reductions in CVD events were observed in the group of subjects aged 40 to 70 years with moderate and high risk, who accounted for 38% of the total cohort and 64% of all CVD events (S14 Table in S1 File). According to age and risk-stratified analyses, the use of S-SCORE enhances cost-effectiveness in elderly and high-risk populations. Incremental QALYs became positive favouring S-SCORE over SCORE in subjects aged >40 years (S16 Table in S1 File). About 87% of all events were observed in the group at baseline age 40 to 70, and 96% of all prevented events was achieved in this group (S2 Fig and S13 Table in S1 File). Less than 9% of population were in the high-risk SCORE category, but S-SCORE generated substantial economic benefits in this group also by withdrawing subjects from continuous medication (S17 Table in S1 File). In summary, subgroup analyses emphasize the necessity for both better risk stratification in population screening and the importance of evaluating targeted approaches in future studies (S19 Table in S1 File).

Our study followed a health system perspective. Data show that beyond direct medical costs for prevention and treatment, CVD imposes a substantial burden of indirect costs to the productivity of societies which is estimated to account for 26% of the total economic costs [68]. The loss in productivity is caused by premature mortality and morbidity related disability affecting engagement in paid work, foreshortening the working life span [69]. One study estimated that using hsTnI for risk stratification in a working-age population avoided productivity losses of €170 per person screened ($230 converted to 2018 Euro) [59]. In our study, 64% of CVD events occurred before the age of 65 years, 40% of which led to premature death before retirement (S15 Table in S1 File). Since the actual indirect costs greatly rely on the social and economic context, we did not calculate the monetary impact but used the potential years of working life lost (PYWLL) as a single indicator for the productivity loss due to CVD related premature death. Stratifying SCORE with hsTnI prevented between 3 and 8 years of productive work time per 1,000 persons screened (Table 4). A few years ago, a study assessed indirect CVD related costs in European countries estimating them to be approximately €210,000 per premature death [70]. Considering this estimate, the reduction in premature deaths with S-SCORE would save indirect costs of €304 per person screened. While an accurate evaluation was beyond the scope of our study, this estimate may additionally support our conclusions. Accounting for the impact of productivity losses from a societal perspective is therefore expected to enhance the preference for S-SCORE.

Comparing the CVD incidence in the study with data from EU countries (5.4% vs. 11.4%, [68]) implies that the cohort used in our study was healthier than a general population in the EU. This is likely caused by differences between participants and non-participants in the underlying cohorts as observed in a large population study in Finland [71]. The subgroup analyses as well as univariate sensitivity analyses for time-to event functions indicate an increase in effectiveness and improvement in cost-effectiveness with increasing baseline risk. Therefore, results presented here can be regarded as conservative, and a higher incidence of events in the general population is expected to further strengthen the preference for the alternative S-SCORE strategy.

At the time when our study was conducted, the updated SCORE (SCORE-II) had not yet been published [72]. While it has been shown that the addition of hsTnI to SCORE improves the prediction of cardiovascular death and cardiovascular disease [22], this information has not yet been evaluated for SCORE-II. Therefore, the current study considers SCORE. Conducting studies in different cohorts, countries, and risk assessment approaches should however be considered in future studies to confirm our findings.

As all decision-analytic modeling studies, our study has several limitations and is based on several assumptions. The study was informed by individual data from multiple longitudinal studies. Characteristics and time-to-events were directly derived from this cohort. Inclusion criteria for the underlying studies may have led to selection bias. We used an age-independent average time-to-event distribution for the cohort stratified by sex and type of CVD event. This could lead to biased results if there are non-linearities in outcomes. For this reason, we varied time-to-event parameters, post event mortality and time horizon in sensitivity analyses but could not find any substantial effect on the ICER. The model follows persons through simplified health states. It also captures time and occurrence of two main cardiovascular events. Multiple events were not considered. The study considered one-time screening at baseline only. Most health services recommend recurring health checks, for example, every five years [7, 66]. An important limitation of our analysis was that the simulation was truncated after ten years in alignment to the accuracy of the ESC risk prediction. Investments in prevention may not fully pay-off within the time span of one decade and further life-years gained would lead to more QALYs than during the restricted 10-year time horizon. It is therefore likely that benefits and cost-effectiveness of S-SCORE is even better than in our base-case analysis. This is supported by scenario analyses of extending the time horizon to 15 years. It is important to emphasize that actual information on treatment and management was not available from the underlying cohort. Also, observed event rates do not necessarily reflect the situation under the SCORE strategy. Consequently, the focus should be on the relative effect of S-SCORE versus SCORE, and absolute number should be used with caution. The treatment effect used in our study indicated the effect of targeting one risk factor with statins. Other drugs such as blood pressure lowering drugs or potentially multiplicative effects of multiple drugs were not considered. Persons frequently have multiple risk factors. Targeting those may lead to optimized prevention. Simulations in our study did not account for potential effects from lifestyle advice or medication adherence although such aspects have been described to be relevant [73, 74]. For adherence, it was assumed that statin effects already accounted for imperfect adherence in an intention-to treat approach. Concurrent lifestyle interventions are expected to reduce the cost per QALY [37]. In our study, 26.6% were smokers. Smoking interventions known to be highly cost-effective were not considered in our analysis. In general, it should however be noted that the relative risk used in the model, although derived from statin, may also describe a more generic effect caused from any other intervention. Previous studies demonstrated that resources used for CVD prevention range considerably between European countries, and cost-effectiveness for specific programs may vary depending on costs of care and prevalence [37, 42, 43, 75]. Therefore, applying conclusions from this study at a specific country level requires caution. While we suggested a sequential approach in S-SCORE, it is worth mentioning that a different algorithm or application of hsTnI alone or together with SCORE is expected to impact results. In addition, clinical decisions based on different CVD prediction tools can vary substantially [76]. One additional note of caution could be that a recent WHO report questioned the effectiveness of systematic population-level screening programs for reducing the burden of cardiovascular diseases in pre-clinical settings. Given these uncertainties, we emphasize the exploratory nature of our study and point out that an optimal strategy cannot be derived. Applying hsTnI information in different schemes or to different risk scores will likely change the outcome and would therefore deserve further research. Also, comparing S-SCORE in future studies to no-testing strategies where all individuals or no one is given medication may help to put results into perspective.

In this study, we evaluated how the addition of hsTnI test results to the guideline-recommended risk assessment could change preventive management and we studied the consequences. As expressed above, the study results are likely to be conservative. However, and in light of some limitations in the underlying cohort and application and management schemes that are still under discussion, we emphasize the exploratory nature of our study. Confirming findings with different cohorts and empirical studies would be worthwhile. For future research, decision-analytic modeling comparing the different options can also be used to find the optimal risk threshold for a novel risk score, an optimal management strategy, or optional screening policies regardless of the current recommendations. A QALY-based health benefit analysis could be performed to inform clinical guidelines and cost-effectiveness analysis could inform reimbursement decisions.

## Conclusions

With an increasing focus on individualizing care pathways, biomarkers such as high sensitivity troponin, which reflect heart specific structural and functional alterations, play an increasing role in improving CVD risk assessment tools. In the context of CVD prevention programs, high sensitivity troponin assessment can have a positive impact on clinical decision making and could therefore be a useful adjunct to existing risk assessment methods for guiding subjects to an appropriate level of preventive care. In addition to improved risk prediction, targeted approaches that stratify SCORE risk classes for future CVD events with hsTnI can lead to changes in management, positively affecting health outcome, and provides a cost-effective alternative strategy particularly in targeted approaches.

## Supporting information

S1 File (PDF)
