# Supplementary material for: Cost-effectiveness of applying high-sensitivity troponin I to a score for cardiovascular risk prediction in asymptomatic population
Source: PLoS One. 2024 Jul 19;19(7):e0307468. doi: 10.1371/journal.pone.0307468 (PMC11259308; doi:10.1371/journal.pone.0307468)
Supplement: S1 File — (PDF) [file pone.0307468.s001.pdf]

S1 File

Cost-effectiveness of applying high-sensitivity troponin I to a score for cardiovascular risk prediction in asymptomatic population

Supplementary material

Box S 1. Overview about the participated cohort studies. 2
Box S 2. Treatment probability and change in management. 4
Table S 1. Acronyms and Abbreviations. 5
Table S 2. BiomarCaRE dataset. 6
Table S 3. Baseline characteristics before and after exclusion. 7
Table S 4. Time to event parametrization derived from the model cohort. 8
Fig S 1. Individual sampling strategy. 9
Table S 5. Model validation (base-case analysis). 10
Table S 6. 2x2 contingency table of concordance of treatment between SCORE and S-SCORE. 11
Table S 7. Impact of S-SCORE on preventive management. 11
Table S 8. Probabilistic sensitivity analysis results. 12
Table S 9. Incremental cost-effectiveness plot report. 12
Table S 10. Frequency of sampling in the microsimulation. 13
Table S 11. Comparison of base-case and microsimulation analysis results. 14
Table S 12. Distribution of subjects by age and SCORE risk category. 15
Table S 13. Number of subjects and CVD events by age and strategy. 16
Table S 14. Number of subjects and CVD events by age, and SCORE risk category, and strategy. 17
Table S 15. Clinical outcomes by strategy. 18
Fig S 2. CVD events by age groups. 19
Table S 16. Subgroup analysis by gender and age. 20
Table S 17. Subgroup analysis by risk classes. 21
Table S 18. Treatment probability in the Base Case and the Derived Management scenario. 22
Fig S 3. Relative risk reduction and number needed to screen in different subgroups and scenarios. 23
Table S 19. Results for S-SCORE vs. SCORE in different subgroups and scenarios. 24
Fig S 4. Change in incremental net monetary benefit (INMB) by varying input assumptions. 25
Fig S 5. Cumulative failure probabilities over 10-year follow-up. 26
References 27

Box S 1. Overview about the participated cohort studies.

Study/cohort	Country	Study/cohort full name and brief description
DanMONICA Study, RCPH(1)	Denmark	The DanMONICA cohorts from the Research Center for Prevention and Health (RCPH) are three prospective population-based cohorts from 11 municipalities from the western part of the suburbs of Copenhagen, Denmark. Random sampling was based on the national population register, stratified by sex and year of birth. Cohort 1 and 3 consists of men and women aged 30-70 years and cohort 2 consists of men and women aged 30-60. Cohort 1 was collected in 1982-1984 (N=4052). Cohort 2 (N=1504) was examined in 1986-1987 and cohort 3 (N=2026) was examined in 1991-1992. Follow-up is achieved through linkage to the National Cause of Death Register and National Hospital Discharge Register, with endpoint diagnosis based on MORGAM criteria and validation described elsewhere. The follow-up for the cohorts 1, 2, and 3 were completed to December 31st, 2010. http://www.thl.fi/publications/morgam/cohorts/full/denmark/den-gloa.htm
KORA, MONICA(2)	Germany	The WHO Multinational Monitoring of Trends and Determinants in Cardiovascular Diseases (MONICA)/ Cooperative Health Research in the Region of Augsburg (KORA) cohorts comprise all respondents from representative sample surveys from the city of Augsburg and the less urban Landkreis Augsburg and Landkreis Aichach-Friedberg regions in Bavaria, Southern Germany. A list of municipalities and population registers was used as sampling frame for the first and the second stage of two-stage sampling, respectively. The second stage of sampling was stratified by sex and ten-year age groups. The Surveys 1 (S1) (baseline: 1984/85; n=4022), S2 (baseline: 1989/90; n=4940) and S3 (baseline: 1994/1995; n=4856) were carried out as part of the WHO MONICA project and S4 (baseline: 1999-2001; n=4261) was carried out within KORA. Participants were aged 25-64 (S1) and 25-74 years (S2-S4) at baseline. Response rates ranged from 79% (S1) to 66% (S4). Follow-up questionnaires were sent to the participants in 2002 and 2009. Coronary events within the study area occurring at ages below 75 years were identified through the MONICA/KORA Augsburg coronary event registry.[13] Non-fatal coronary events which occurred outside the study area and in participants aged ≥75 years and all incident strokes were identified by questionnaires. Mortality follow-up until 2009 was conducted through national death registers and fatal coronary events and fatal strokes were identified through death certificates. Coronary events and strokes were validated by autopsy reports, death certificates or medical records. Information on incident stroke is only available for S3 and S4 in the MORGAM database, therefore, the composite variable on incident CVD is also only available for S3 and S4. The present analysis used only data from the S3 and S4 studies. http://www.thl.fi/publications/morgam/cohorts/full/germany/ger-auga.htm
Northern Sweden MONICA Study(3)	Sweden	The Northern Sweden cohort was recruited in the Västerbotten and the Norrbotten counties. Both counties are a sparsely populated area, half rural and half urban with higher-than-average mortality and unemployment and low socio-economic status. The cohort was formed by the respondents of representative sample surveys with an age from 24 to 75. National population register was used as sampling frame for the single stage sampling which was stratified by sex and 10-year age group. The baseline examinations were partly carried out as part of the WHO MONICA Project. Recruitment was performed in 1986 (N=1,625), 1990 (N=1,576), 1994 (N=1,893), 1999 (N=1,789), 2004 (N=1,863) and 2009 (N=1,704). The response rate was between 68 and 81%. Everyone in Sweden has a unique personal identification code issued by the National Tax Authority. The cohorts were linked to the registers mentioned above using the personal identification code, all dates of death, and the causes of death were obtained until the 31st of December 2011. http://www.org.umu.se/monica https://www.thl.fi/publications/morgam/cohorts/full/sweden/swe-nswa.htm
MATISS Rome Study(4)	Italy	Participants of the MATISS (Malattie cardiovascolari ATerosclerotiche, Istituto Superiore di Sanità [Italian]) study were recruited in the district of Latina between 1983 and 1987 (N=8,265) and 1993-1996 (N=1,970). Electoral rolls were used as sampling frames in the single-stage sampling, which was stratified by the municipality, sex and 5-year age group. The baseline examination was partly carried out as part of the WHO MONICA Project, where it was identified with code ITA-LAT. The other examinations were carried out as part of the MATISS Project using the WHO MONICA methods. Individuals were 20 to 69 years old. From the first screening (1983) to the last screening (1996), municipalities were contacted every five years for information about vital status, emigration and residency; from 1996 onwards, municipalities were contacted every year. The follow-up procedure covers the Lazio Region. If a person moved out of the Lazio Region, then he/she was lost to follow-up since the date of emigration. Follow-up was completed to 31st December 2004. https://www.thl.fi/publications/morgam/cohorts/full/italy/ita-roma.htm

Moli-sani Study(5)	Italy	The cohort of the Moli-sani study was recruited in the Molise region from city hall registries by multistage sampling. First, townships were sampled in major areas by cluster sampling; then, within each township, participants aged 35 years or older were selected by simple random sampling. Exclusion criteria were pregnancy at the time of recruitment, inability to comprehend, current multiple trauma or coma, or refusal to sign the informed consent. A total of 24 325 men (47%) and women (53%) were examined at baseline from 2005 to 2010. Participation was 70%. The cohort was followed up to December 2011. Follow-up is achieved through record linkage to national mortality registries and hospital discharge registers, validation of events was achieved through hospital record linkage and doctor's medical records using updated MORGAM criteria. http://www.moli-sani.org/
MONICA Brianza Study(6)	Italy	The MONICA-Brianza Cohort Study is a prospective observational study of three cohorts of 25-64 years old residents in Brianza, a highly-industrialized area located between Milan and the Swiss border, Northern Italy. Gender- and ten-year age-stratified samples were randomly drawn in 1986, 1990, and 1993, and cardiovascular risk factors were investigated at baseline following the procedures of the WHO MONICA Project. The overall participation rate was 69%. For all subjects whole-blood and serum samples were stored in a biobank. The protocol was approved by the Monza Hospital Ethical Committee. Study participants were followed up for first coronary or stroke events, fatal and non-fatal, up to the end of 2008, for a median of 15 years. http://epimed.uninsubria.eu
FINRISK(7)	Finland	The FINRISK study is a series of population-based cardiovascular risk factor surveys carried out every five years in five (or six in 2002) districts of Finland, including North Karelia, Northern Savo (former Kuopio), Southwestern Finland, Oulu Province, Lapland province (in 2002 only), and the region of Helsinki and Vantaa. A stratified random sample was drawn for each survey from the national population register; the age-range was 25-74 years. All individuals enrolled in the study received a physical examination, a self-administered questionnaire, and a blood sample was drawn. FINRISK cohorts based on surveys carried out in 1982, 1987, 1992, 1997 and 2002 were used in this analysis. The numbers of participants in each survey are shown in **Table S2** and the participation rates can be found in the web address below. During follow-up, the National Hospital Discharge Register, the National Causes of Death Register and the National Drug Reimbursement Register were used to identify endpoints. In these analyses, the follow-up extends until Dec. 31st, 2010. The Coordinating Ethics Committee of the Helsinki and Uusimaa Hospital District approved the study, which followed the declaration of Helsinki. All subjects gave informed consent. http://www.thl.fi/publications/morgam/cohorts/full/finland/fin-fina.htm
PRIME(8)	United Kingdom	The PRIME (Prospective Epidemiological Study of Myocardial Infarction) study examined the classic and putative cardiovascular risk factors to explain the large difference in heart disease incidence between Ireland and France. The study includes four cohorts of men aged 50-59; from Belfast, Northern Ireland (N=2,745) and Lille (N=2,633), Toulouse (N=2,610) and Strasbourg (N=2,612) in France. Baseline examinations took place in 1990-1993 and targeted cohorts which had broadly similar social class structures to the background population, initially sampling from industries and various employment groups, employment groups with more than 10% of their workforce of foreign origin were excluded. Follow-up for 10 years for each participant (Toulouse, Strasbourg and Lille) and for 18 years (Belfast) was achieved through annual follow up questionnaires with verification against national death registers, medical records, hospital discharge diagnoses. Endpoints were validated by expert medical committee. http://www.thl.fi/publications/morgam/cohorts/full/uk/unk-bela.htm
SHHEC(9)	Scotland	The Scottish Heart Health Extended Cohort (SHHEC) comprises different cohorts of men and women aged 25-64 (25-75 in SHHEC 2) recruited randomly across Scottish districts from 1984-1995 in the Scottish Heart Health Study and Scottish MONICA in contribution to the WHO MONICA Project. Apart from age differences the surveys following the same protocol. Of 18,107 in SHHEC, those 15,999 with the most complete risk factor data were entered into the MORGAM study and thence the MORGAM Biomarker Study and BiomarCaRE. Follow-up data extends to the end of 2009 using the Scottish National Health Service Central Register and the Scottish Record Linkage System for mortality and cardiovascular endpoints. https://www.thl.fi/publications/morgam/cohorts/full/uk/unk-sco.htm

Box S 2. Treatment probability and change in management.

Treatment eligibility or ineligibility in our study is based on guideline recommendations for SCORE risk categories. As our study aimed to simulate a potential change in management (aka individuals who receive or do not receive preventive therapy), we had to go beyond eligibility. Therefore, we assumed and assigned specific treatment probabilities that were based on the recommended eligibility.

As an example (see Table 2):

- SCORE High risk (>=5% and <10%)
  - Recommendation: "may be candidates for drug treatment" (ESC guidelines 2016)
  - Assumed treatment probability: 50% (30-75%)

We further assumed that the additional information obtained by hsTnI would change this treatment probabilities: An elevated hsTnI would increase, a low hsTnI would decrease the probability for treatment.

- SCORE High risk <u>plus</u> hsTnI- (F: <4, M: <6ng/L)
  - Decreased assumed treatment probability: 30% (1-50%)
- SCORE High risk <u>plus</u> hsTnI+ (F: 4-10, M: 6-12ng/L)
  - Increased assumed treatment probability: 75% (50-99%)

The estimated "Change in management" is therefore the result of specific cohort characteristics and the difference in the assumed treatment probabilities between the two strategies SCORE and S-SCORE.

**Treatment assignment in SCORE**	**Treatment assignment in S-SCORE**	**Change in management**	**Risk in SCORE**	**Risk in S-SCORE compared to SCORE**
No treatment	No treatment	No change	Standard risk	Standard risk
No treatment	Treatment	Change	Standard risk	Reduced risk
Treatment	No treatment	Change	Standard risk	Increased risk
Treatment	Treatment	No change	Standard risk	Standard risk

Table S 1. Acronyms and Abbreviations.

BC	Base case scenario
CHD	Coronary heart disease
CVD	Cardiovascular disease
DM	Derived management scenario
HSU	Health state utilities
hsTnI	High-sensitivity troponin I
ICER	Incremental cost-effectiveness ratio
INMB	Incremental net monetary benefit
SCORE	Systematic Coronary Risk Evaluation
S-SCORE	Stratified SCORE using hsTnI
QALY	Quality adjusted life year
WTP	Willingness-to-pay

Table S 2. BiomarCaRE dataset.

Participating center	Country	Before exclusion	After exclusion (Model cohort)	Baseline survey	Last year of follow-up
Augsburg	Germany	8,842	5,841	1984-2001	2009
Brianza	Italy	4,932	3,078	1986-1993	2008
DAN-MONICA	Denmark	7,582	6,858	1982-1992	2010
FINRISK	Finland	8,444	6,807	1982-2002	2010
Moli-sani	Italy	24,325	22,170	2005-2010	2011
Northern Sweden	Sweden	10,450	9,441	1986-2009	2011
PRIME/Belfast	Ireland	2,745	2,010	1990-1993	2011
Rome	Italy	4,489	3,364	1983-1996	2004
Scotland	Scotland	15,999	12,621	1984-1995	2009
**Total**		**87,808**	**72,190**	**1982-2010**	**2011**

Individuals with missing values in the variables required to compute SCORE, hsTnI or the CVD follow-up variables, individuals with examination age < 20 or > 85 years, and individuals with a CVD history at baseline were excluded from the baseline dataset.

Table S 3. Baseline characteristics before and after exclusion.

	Before exclusion			After exclusion (Model cohort)		
	All (N=87808)	Men (N=44344)	Women (N=43464)	All (N=72190)	Men (N=35173)	Women (N=37017)
Examination age (years)	51.0 (41.6, 59.8)	51.6 (42.1, 59.9)	50.4 (41.1, 59.6)	50.6 (41.3, 59.2)	50.9 (41.7, 59.2)	50.1 (41.0, 59.2)
	NAs: 0	NAs: 0	NAs: 0	NAs: 0	NAs: 0	NAs: 0
Survey year, range	1982-2010	1982-2010	1982-2010	1982-2010	1982-2010	1982-2010
	NAs: 0	NAs: 0	NAs: 0	NAs: 0	NAs: 0	NAs: 0
Male No. (%)	44344 (50.5)	44344 (100)	0 (0)	35173 (48.7)	35173 (100)	0 (0)
	NAs: 0	NAs: 0	NAs: 0	NAs: 0	NAs: 0	NAs: 0
BMI (kg/m²)	26.2 (23.6, 29.4)	26.6 (24.3, 29.2)	25.8 (22.9, 29.6)	26.2 (23.5, 29.3)	26.5 (24.3, 29.1)	25.7 (22.8, 29.5)
	NAs: 263	NAs: 116	NAs: 147	NAs: 0	NAs: 0	NAs: 0
Systolic BP (mmHg)	131.5 (119.0, 147.0)	134.0 (122.0, 148.0)	129.0 (116.0, 145.0)	131.0 (119.0, 146.0)	133.5 (122.0, 147.0)	128.0 (116.0, 145.0)
	NAs: 100	NAs: 52	NAs: 48	NAs: 0	NAs: 0	NAs: 0
Total cholesterol (mmol/L)	5.7 (5.0, 6.5)	5.7 (5.0, 6.5)	5.7 (5.0, 6.6)	5.7 (5.0, 6.5)	5.7 (5.0, 6.5)	5.7 (5.0, 6.5)
	NAs: 328	NAs: 163	NAs: 165	NAs: 0	NAs: 0	NAs: 0
Diabetes No. (%)	3787 (4.3)	2151 (4.9)	1636 (3.8)	2792 (3.9)	1502 (4.3)	1290 (3.5)
	NAs: 365	NAs: 175	NAs: 190	NAs: 304	NAs: 144	NAs: 160
Daily smoker No. (%)	23330 (26.7)	12847 (29.2)	10483 (24.2)	19046 (26.4)	10088 (28.7)	8958 (24.2)
	NAs: 493	NAs: 283	NAs: 210	NAs: 0	NAs: 0	NAs: 0

If not otherwise stated, continuous variables are presented as median, 25th percentile, and 75th percentile. Binary variables are described as absolute and relative frequencies. Missing information is shown as NA (Not available).

Table S 4. Time to event parametrization derived from the model cohort.

Event in 10YU	Cohort	Survey year, Range	AGE at examination, years, Range	Age at end of FU, years, Range	FU time, years, Range	Number of individuals	Number of events	FU time, years			Weibull parameter		% event 10Y	
								25th Perc.	Median	75th Perc.	Shape k, (95%CI)	Scale λ (95%CI)	KM	Wb
CHD	Men	1982-2010	20.6 - 84.9	24.2 – 93.0	0 - 28.2	35,173	3,357	5.1	12.9	18.0	1.227617 (1.183924; 1.279422)	0.0040631 (0.0035352; 0.0045755)	6.5	6.6
	Women	1982-2010	21.3 – 85.0	24.0 - 92.8	0 - 28.2	37,017	1,494	5.0	10.8	18.8	1.475561 (1.386064; 1.573681)	0.0008149 (0.0006352; 0.0010293)	2.2	2.4
Stroke	Men	1982-2010	20.6 - 84.9	24.2 – 93.0	0 - 28.2	35,173	700	4.9	11.3	18.0	1.295609 (1.172743; 1.426938)	0.0006907 (0.0004865; 0.0009462)	1.3	1.4
	Women	1982-2010	21.3 – 85.0	24.0 - 92.8	0 - 28.2	37,017	480	5.0	10.6	17.9	1.412400 (1.267330; 1.573561)	0.0003208 (0.0002110; 0.0004554)	0.7	0.8
Death	Men	1982-2010	20.6-84.9	24.2-93	0-28.2	35173	3305	5.0	12.8	18.0	1.410594 (1.359159; 1.468755)	0.0023747 (0.0020403; 0.0027279)	5.8	5.9
	Women	1982-2010	21.3-85	24-92.8	0-28.2	37017	2339	5.1	11.0	19.5	1.539657 (1.460338; 1.617952)	0.0010434 (0.0008380; 0.0012808)	3.4	3.6

Shape k and Scale λ are the corresponding Weibull parameters of the Weibull distribution fitted to the time-to-event data using the parametrization used by R-function rweibull and transformed into the Weibull from of the modelling software using the following parametrization for the density function: f(x)= λkx^(k-1) e^(-λx^k). The column % event 10y KM was computed using the Kaplan-Meier estimator. The column % event 10y Wb was computed assuming a Weibull distribution for the time-to-event variables. 95% confidence intervals were estimated from 1,000 bootstrapped samples.

Fig S 1. Individual sampling strategy.

In the base-case analysis, 20,000 individual samples were drawn. This was repeated 100 times in independent runs to obtain the expected values. The results based on the standard risk of the SCORE strategy was validated against observed event rates in the BiomarCaRE study cohort.

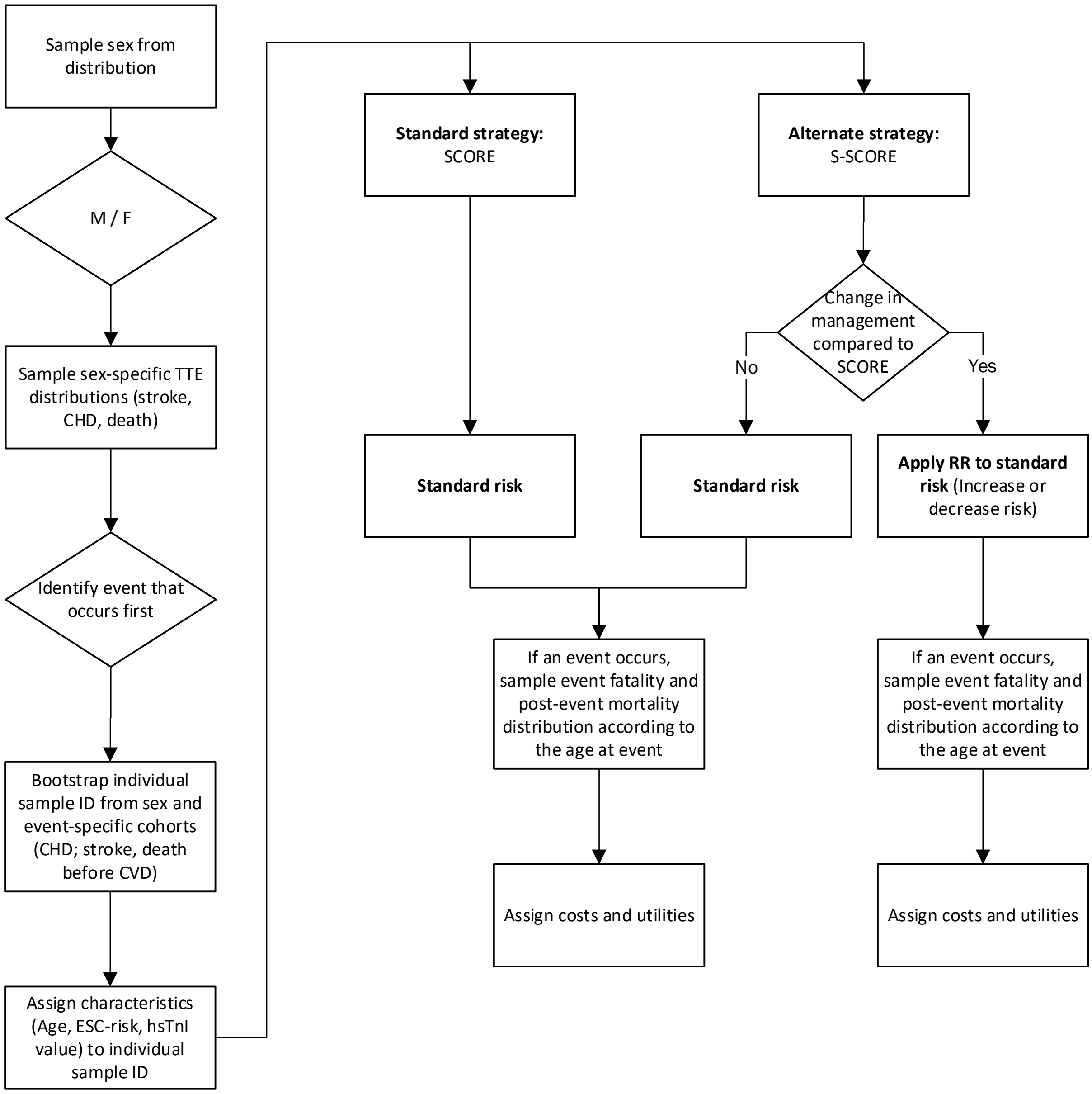

Table S 5. Model validation (base-case analysis).

Outcome in 10YFU	BMC Cohort	Model	
	KM	Mean	95%CI
CVD Total, %	5.3	5.36	(5.33 - 5.39)
CHD, %	4.3	4.34	(4.31 - 4.37)
Stroke, %	1.0	1.02	(1.01 - 1.04)
Death before CVD, %	4.6	4.66	(4.62 – 4.69)

Cardiovascular disease event rates in 10 years of follow-up as observed in the study cohort (BMC cohort) and as calculated by the model for the standard strategy (SCORE). For validation, results reflect 100 runs each randomly sampling 20,000 individuals from the study cohort. KM: Kaplan-Meier estimator.

Table S 6. 2x2 contingency table of concordance of treatment between SCORE and S-SCORE.

		SCORE (Standard strategy)		
		No treatment, %	Treatment, %	SUM (S-SCORE), %
S-SCORE (alternative strategy)	No treatment, %	83.0	2.5	85.5
	Treatment, %	7.5	7.0	14.5
	SUM (SCORE), %	90.5	9.5	100

Table S 7. Impact of S-SCORE on preventive management.

Category	Total, % of all	Treated % of all in category		Impact of S-SCORE, % of all in category		Direction of change, % of all in category	
		SCORE	S-SCORE	No change	Change	Off treatment	To treatment
All	100.0	9.5	14.5	90.0	10.0	2.5	7.5
Age 40-70, HMR	37.7	8.6	20.7	78.0	22.0	5.0	17.1
By gender							
Female	51.5	6.0	10.8	92.0	8.0	1.6	6.4
Male	48.5	13.1	18.4	87.8	12.2	3.5	8.8
By SCORE risk class							
Low (<1%)	54.2	0.0	0.7	99.3	0.7	0.0	0.7
Moderate (1 to <5%)	31.9	0.0	16.5	83.5	16.5	0.0	16.5
High (5 to <10%)	8.7	50.4	42.7	50.2	49.8	28.7	21.1
Very high (>10%)	5.1	98.8	100.0	98.8	1.2	0.0	1.2
By age group							
20-30	5.5	0.0	0.9	99.1	0.9	0.0	0.9
31-40	15.8	0.0	0.7	99.3	0.7	0.0	0.7
41-50	28.3	0.1	2.6	97.4	2.6	0.0	2.6
51-60	28.8	3.6	14.7	86.0	14.0	1.5	12.5
61-70	15.5	25.8	31.8	75.6	24.4	9.2	15.2
71-80	5.7	68.8	69.5	76.9	23.1	11.2	11.9
81-85	0.5	95.8	97.2	96.2	3.8	1.2	2.6
40-70	76.9	10.1	13.6	87.8	12.6	2.6	9.2

Results from microsimulation analysis with 250,000 samples S-SCORE vs. SCORE. HMR: High and moderate SCORE risk.

Table S 8. Probabilistic sensitivity analysis results.

Outcome	SCORE		S-SCORE		Incremental values			
	Mean	95%CI	Mean	95%CI	Mean	95%CI	p-value	T-value
Preventive treatment, %	9.43	(9.39; 9.48)	14.48	(14.44; 14.53)	5.05	(4.99; 5.11)	0.000	157
Changed management vs. SCORE, %			10.06	(10.02; 10.10)				
CVD, %	5.36	(5.34; 5.38)	4.85	(4.83; 4.87)	-0.51	(-0.54; -0.48)	0.000	-32
CHD, %	4.33	(4.31; 4.35)	3.89	(3.87; 3.91)	-0.43	(-0.46; -0.41)	0.000	-30
Stroke, %	1.03	(1.03; 1.04)	0.96	(0.95; 0.96)	-0.08	(-0.09; -0.07)	0.000	-16
All-cause Mortality, %	7.11	(7.07; 7.16)	6.87	(6.82; 6.91)	-0.24	(-0.31; -0.02)	0.000	-7
CVD related Mortality, %	2.37	(2.35; 2.38)	2.13	(2.12; 2.14)	-0.24	(-0.26; -0.22)	0.000	-25
EFS, years p. 1,000 pers.	9,570	(9,568; 9,572)	9,592	(9,590; 9,594)	22	(19; 25)	0.000	15
PYWLL per 1,000 pers.	116	(115; 117)	111	(110; 112)	-5	(-6; -4)	0.000	-7
Costs, €	1,350	(1,342; 1,358)	1,539	(1,531; 1,548)	189	(177; 201)	0.000	32
QALYs (x 1,000)	8,212	(8,210; 8,214)	8,218	(8,217; 8,220)	6	(4; 9)	0.000	6
ICER					33,942	(10,238; 126,165)		

Results from probabilistic sensitivity analysis with 500 repetitions of microsimulations with 20,000 individuals. EFS: Years of event-free survival. PYWLL: Potential years of working life lost assuming a retirement at 65years. QALY: Quality adjusted life years. ICER: Incremental cost-effectiveness ratio. ICER confidence intervals estimated from 2.5th and 97.5th percentile of ICER distribution.

Table S 9. Incremental cost-effectiveness plot report.

Component	Quadrant	Incr. Eff.	Incr. costs	ICER	Frequency	Proportion
C1	IV	IE>0	IC<0	Superior	0	**0.0**
C2	I	IE>0	IC>0	ICER<WTP	400	**80.0**
C3	III	IE<0	IC<0	ICER>WTP	0	0.0
C4	I	IE>0	IC>0	ICER>WTP	93	18.6
C5	III	IE<0	IC<0	ICER<WTP	0	0.0
C6	II	IE<0	IC>0	Inferior	7	1.4
Indifferent	origin	IE=0	IC=0	0/0	0	0.0

Number of iterations per result category from probabilistic sensitivity analysis (PSA) (n=500). Scatterplot graph is shown in Fig 2.

Table S 10. Frequency of sampling in the microsimulation.

Frequency of sampling	N (Total 72,190)	%
0	2,903	4.0
1	8,324	11.5
2	13,364	18.5
3	14,937	20.7
4	12,754	17.7
5	9,156	12.7
6	5,510	7.6
7	2,990	4.1
8	1,373	1.9
9	578	0.8
10	198	0.3
11	68	0.1
12	23	0.0
13	9	0.0
14	1	0.0
15	2	0.0

By randomly sampling 250,000 individuals in a microsimulation, about 4% of cases remained unconsidered in the study.

Table S 11. Comparison of base-case and microsimulation analysis results.

Outcome	Sampling	Mean	(95% CI)	Q1	Median	Q3
**Incremental costs**	BC	187	(177; 196)	173	188	199
	MS	185	(155; 215)	25	25	25
**Incremental QALY (x1,000)**	BC	7	(5; 9)	5	7	9
	MS	8	(2; 13)	0	0	0
**ICER**	BC	27,440	(13,429; 123,027)	19,696	27,894	40,584
	MS	23,125	n/a	n/a	n/a	n/a
**INMB**	BC	153	(45; 262)	45	136	270
	MS	192	(-109; 495)	-25	-25	-25

Microsimulation (MS): 250,000 individual samples. Base-case (BC): 100 repetitions of microsimulations with 20,000 individual samples. Statistics refer to individual distribution (MS) or average of the mean (BC). ICER: Incremental cost-effectiveness ratio. INMB: Incremental net monetary benefit assuming a willingness-to-pay threshold of 50,000 €. Confidence interval for ICER estimated from 2.5th and 97.5th percentile of distribution. ICER statistics of MS not available since denominator different from zero in only 10% of cases.

Table S 12. Distribution of subjects by age and SCORE risk category.

	Number of subjects, N=250,000 (% of all)							
	Female: 128,859 (51.5%)				Male: 121,141 (48.5%)			
Age	Low	Moderate	High	Very High	Low	Moderate	High	Very High
20-30	7,427 (3.0)	0 (0.0)	0 (0.0)	0 (0.0)	6,203 (2.5)	0 (0.0)	0 (0.0)	0 (0.0)
31-40	20,785 (8.3)	0 (0.0)	0 (0.0)	0 (0.0)	18,502 (7.4)	88 (0.0)	0 (0.0)	0 (0.0)
41-50	37,523 (15.0)	281 (0.1)	0 (0.0)	0 (0.0)	22,614 (9.0)	10,292 (4.1)	86 (0.0)	6 (0.0)
51-60	19,865 (7.9)	14,582 (5.8)	208 (0.1)	20 (0.0)	2,275 (0.9)	30,536 (12.2)	3,863 (1.5)	547 (0.2)
61-70	283 (0.1)	15,321 (6.1)	3,648 (1.5)	725 (0.3)	0 (0.0)	7,307 (2.9)	8,186 (3.3)	3,348 (1.3)
71-80	0 (0.0)	1,191 (0.5)	3,589 (1.4)	2,739 (1.1)	0 (0.0)	236 (0.1)	2,189 (0.9)	4,209 (1.7)
81-85	0 (0.0)	0 (0.0)	49 (0.2)	623 (0.2)	0 (0.0)	0 (0.0)	18 (0.0)	636 (0.3)

Results from microsimulation analysis with 250,000 samples.

Table S 13. Number of subjects and CVD events by age and strategy.

	Subjects		CVD SCORE			CVD S-SCORE		
Age	N	%	N	% CVD	% in Age	N	% CVD	% in Age
20-30	13,630	5.5	49	0.4	0.36	62	0.5	0.46
31-40	39,375	15.8	652	4.8	1.66	672	5.5	1.71
41-50	70,802	28.3	2,667	19.7	3.77	2,543	20.9	3.59
51-60	71,896	28.8	5,597	41.4	7.78	4,895	40.2	6.81
61-70	38,818	15.5	3,521	26.1	9.07	3,048	25.0	7.85
71-80	14,153	5.7	975	7.2	6.89	920	7.5	6.50
81-85	1,326	0.5	50	0.4	3.77	50	0.4	3.77
Sum	250,000		13,511			12,190		
%			5.4			4.9		

Results from microsimulation analysis with 250,000 samples.

Table S 14. Number of subjects and CVD events by age, and SCORE risk category, and strategy.

	Subjects				Number of CVD events							
					SCORE				S-SCORE			
Age	Low	Moderate	High	Very High	Low	Moderate	High	Very High	Low	Moderate	High	Very High
20-30	13,630	0	0	0	49	0	0	0	62	0	0	0
31-40	39,287	88	0	0	641	11	0	0	657	15	0	0
41-50	60,137	10,573	86	6	1,658	983	26	0	1,665	860	18	0
51-60	22,140	45,118	4,071	567	725	3,987	745	140	732	3,483	539	141
61-70	283	22,628	11,834	4,073	6	1,625	1,295	595	6	1,420	1,024	598
71-80	0	1,427	5,778	6,948	0	85	346	544	0	68	306	546
81-85	0	0	67	1,259	0	0	0	50	0	0	0	50
Sum	135,477	79,834	21,836	12,853	3,079	6,691	2,412	1,329	3,122	5,846	1,887	1,335
%	54.2	31.9	8.7	5.1	22.8	49.5	17.9	9.8	25.6	48.0	15.5	11.0

	Difference in number of CVD events				P-value			
Age	Low	Moderate	High	Very High	Low	Moderate	High	Very High
20-30	13				*0.25*			
31-40	16	4			*0.68*	*0.53*		
41-50	7	-123	-8	0	*0.92*	**0.00**	*0.22*	*1.00*
51-60	7	-504	-206	1	*0.87*	**0.00**	**0.00**	*1.00*
61-70	0	-205	-271	3	*1.00*	**0.00**	**0.00**	*0.95*
71-80		-17	-40	2		*0.18*	*0.16*	*0.98*
81-85			0	0			*1.00*	*1.00*

Results from microsimulation analysis with 250,000 samples.

Table S 15. Clinical outcomes by strategy.

		SCORE			S-SCORE			Difference		
			Event age			Event age			Event age	
**Variable**	**N**	**N**	**<65**	**>65**	**Events**	**<65**	**>65**	**Events**	**<65**	**>65**
CVD event	250,000	13,511	8,629	4,882	12,190	7,908	4,282	-1,321	-721	-600
Deaths (all causes)	250,000	17,484	3,792	13,691	16,822	3,443	13,379	-662	-349	-312
Deaths (CVD related)	250,000	6,008	3,617	2,391	5,360	3,255	2,105	-648	-362	-286
PYWLL	250,000	30,197	30,197	0	28,434	28,434	0	-1,763	-1,763	0

Results from microsimulation analysis with 250,000 samples.
PYWLL: Potential years of working life lost due to premature death assuming a retirement age of 65 years.

Fig S 2. CVD events by age groups.

Cumulative 10-years risk per age group estimated from microsimulation analysis with 250,000 samples.

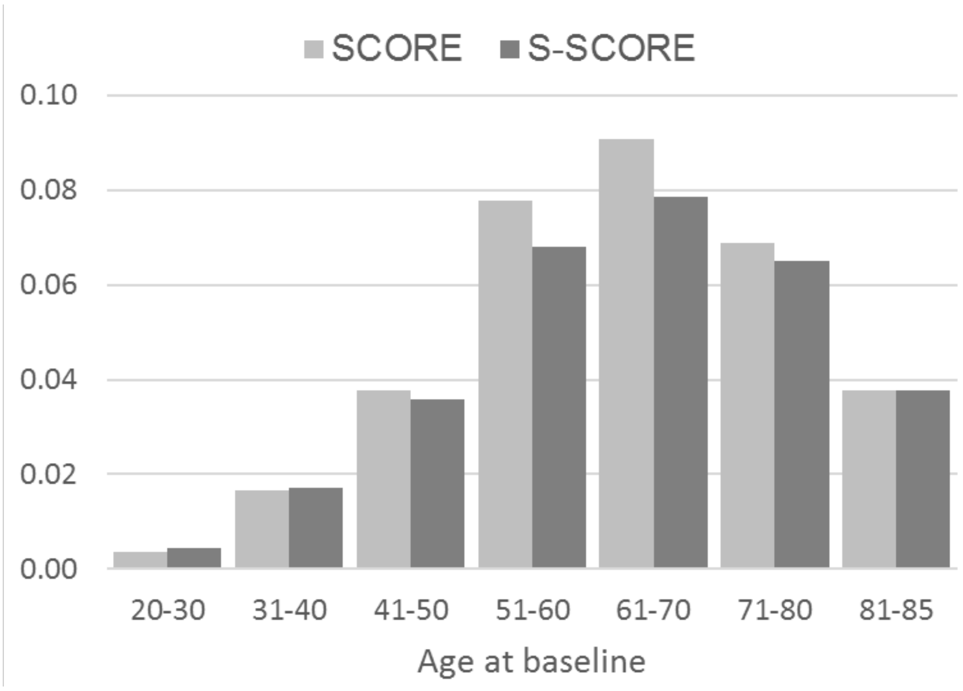

Table S 16. Subgroup analysis by gender and age.

A. Female (N 128,859; 51.5%)

Age	N	Percent	Costs 1	Costs 2	IC	QALY 1	QALY 2	IE (x1,000)	INMB	ICER
All	128859	51.5	886	1081	196	8.3047	8.3089	**4.2**	**14.5**	**46,429**
20-30	7427	5.8	76	143	67	8.4645	8.4638	-0.7	-103	-96,143
31-40	20785	16.1	163	207	44	8.4486	8.4481	-0.5	-65	*-87,800*
41-50	37804	29.3	413	475	61	8.3852	8.3854	0.2	-53	305,500
51-60	34675	26.9	822	1194	372	8.2413	8.2461	**4.8**	-134	77,500
61-70	19977	15.5	1757	2158	401	8.1383	8.1538	**15.5**	**377**	**25,871**
71-80	7519	5.8	3658	3728	70	8.0902	8.0996	**9.4**	**401**	**7,479**
81-85	672	0.5	5119	5191	72	8.1819	8.1881	**6.2**	**236**	**11,613**

B. Male (N 121,141; 48.5%)

Age	N	Percent	Costs 1	Costs 2	IC	QALY 1	QALY 2	IE (x1,000)	INMB	ICER
All	121141	48.5	1867	2041	174	8.1202	8.1313	**11.1**	**381**	**15,676**
20-30	6203	5.1	133	240	106	8.4518	8.4484	-3.4	-279	*-31,294*
31-40	18590	15.3	466	558	93	8.3779	8.3755	-2.4	-212	*-38,708*
41-50	32998	27.2	981	1182	201	8.2666	8.2698	**3.2**	-42.7	62,813
51-60	37221	30.7	2004	2409	405	7.9943	8.0109	**16.6**	**427**	**24,398**
61-70	18841	15.6	3846	3692	**-154**	7.8534	7.8872	**33.8**	**1846**	**-4,556**
71-80	6634	5.5	5082	5048	**-34**	7.8454	7.8532	**7.8**	**424**	**-4,359**
81-85	654	0.5	5385	5521	136	7.9135	7.9087	-4.8	-376	*-28,292*

Microsimulation with 250,000 sampled subjects from the study cohort. Index 1 refers to the standard strategy SCORE. Index 2 refers to the alternative strategy S-SCORE. Net monetary benefits (NMB) assuming a willingness-to-pay threshold per QALY of 50,000€. INMB: Incremental net monetary benefit. Favorable results for Stratified-SCORE compared to SCORE are shown in bold. IC: Incremental costs (S-SCORE – SCORE). IE: Incremental QALYs. ICER: Incremental cost-effectiveness ratio

Table S 17. Subgroup analysis by risk classes.

SCORE risk class	Stratified class	All					Female					Male				
		Proportion	IC	IE *1,000	INMB	ICER	Proportion	IC	IE *1,000	INMB	ICER	Proportion	IC	IE *1,000	INMB	ICER
Low	(-)	48.4	25	0	-25		59.1	25	0	-25		37.1	25	0	-25	
	(+)	4.7	55	-2	-157	-35,738	6.6	66	-2	-167	-32,525	2.7	65	-1	-131	-48,647
	(++)	1.1	1,664	-45	-3,936	-38,418	1.0	1,585	-32	-3,202	-49,071	1.1	1,828	-56	-4,622	-32,701
Moderate	(-)	25.3	25	0	-25		18.5	25	0	-25		32.6	25	0	-25	
	(+)	5.5	2,906	19	-2,021	156,878	5.0	3,023	**19**	-2,061	157,448	6.0	2,914	**19**	-1,986	157,514
	(++)	1.2	3,412	164	**4,973**	**19,609**	0.9	2,704	**207**	**7,635**	**13,075**	1.5	3,538	**135**	**3,227**	**26,149**
High	(-)	6.5	-1,279	53	**3,803**	**-22,328**	3.9	**-1,120**	**31**	**2,652**	**-36,547**	9.2	**-1,198**	**62**	**4,318**	**-19,202**
	(+)	1.8	249	86	**3,772**	**6,319**	1.6	903	**35**	**822**	**26,174**	2.0	227	**133**	**6,410**	**1,711**
	(++)	0.5	1,427	68	**1,930**	**21,479**	0.3	2,283	**51**	**266**	**44,765**	0.7	1,056	**76**	**2,721**	**13,987**
Very High	(-)	3.2	95	0	-97	1,020,000	1.7	92	-2	-206	-40,087	4.9	106	**1**	-56	105,900
	(+/++)	1.9	99	-5	-359	-19,114	1.5	104	-1	-130	-199,615	2.3	96	-8	-512	-11,576

Subgroup analysis derived from microsimulation with 250,000 sampled subjects from the study cohort. Results favouring S-SCORE compared to SCORE are shown in bold. INMB: Incremental net monetary benefit at a willingness-to-pay threshold of 50,000 €. A positive INMB indicates that S-SCORE generates cost-effective results compared to SCORE in the respective subgroup.

Table S 18. Treatment probability in the Base Case and the Derived Management scenario.

Risk class	Probability for preventive treatment	
	Base case (BC)	Derived Management scenario (DM)
SCORE Low	0.00	0.00
S-SCORE Low (-)	0.00	0.00
S-SCORE Low (+)	0.01	0.01
S-SCORE Low (++)	0.30	0.01
SCORE Mod	0.00	0.00
S-SCORE Mod (-)	0.00	0.00
S-SCORE Mod (+)	0.75	0.01
S-SCORE Mod (++)	0.99	0.50
SCORE High	0.50	0.50
S-SCORE High (-)	0.30	0.01
S-SCORE High (+)	0.75	0.50
S-SCORE High (++)	0.99	0.99
SCORE Very high	1.00	1.00
S-SCORE Very high (-)	0.99	0.99
S-SCORE Very high (+/++)	1.00	1.00

Fig S 3. Relative risk reduction and number needed to screen in different subgroups and scenarios.

Scenarios were analyzed by applying the base case sampling approach. Results for subgroups (*) were derived from a microsimulation including 250,000 individuals. DM: Derived Management scenario. NNS: Number needed to screen to prevent one event. RRR: Relative risk reduction.

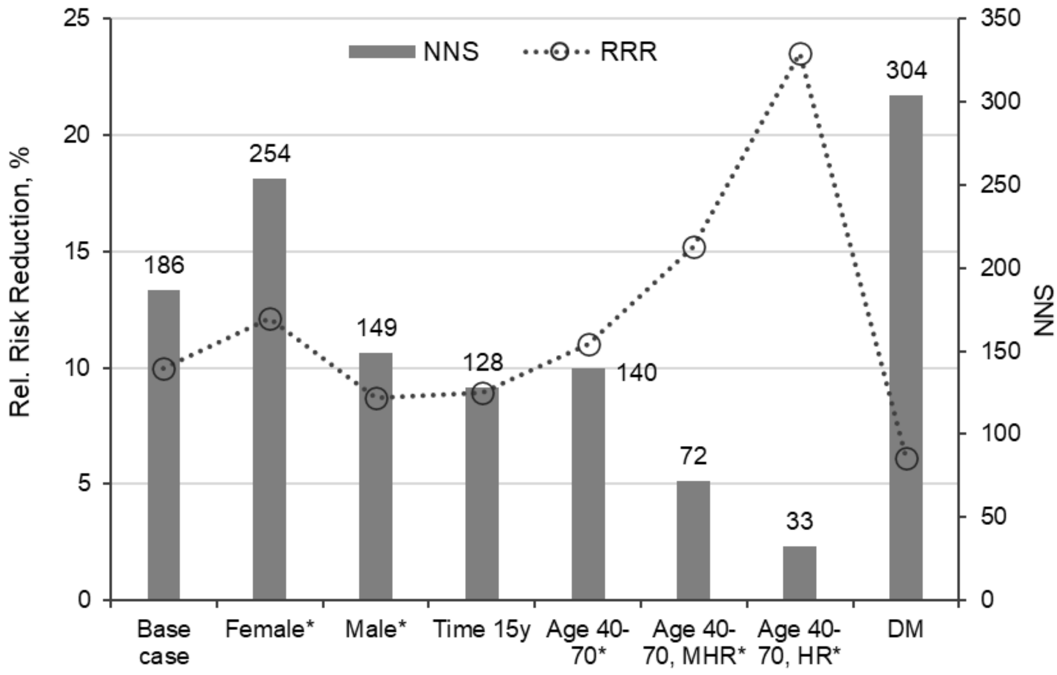

Table S 19. Results for S-SCORE vs. SCORE in different subgroups and scenarios.

Subgroup / Scenario	Treatment, %			Costs, €			QALYs*1000			ICER, €/QALY gained	RRR, %	NNS (95%CI)
	SCORE	S-SCORE	Changed	SCORE	S-SCORE	IC	SCORE	S-SCORE	IE			
Female*	6.0	10.8	8.0	886	1,081	196	8,305	8,309	4.2	46,419	12.1	254 (190; 383)
Base Case scenario	9.4	14.5	10.0	1,349	1,536	187	8,214	8,221	6.8	27,440	10.0	186 (172; 203)
Undiscounted Base Case scenario	9.4	14.4	10.0	1,598	1,805	206	9,468	9,476	8.3	24,727	10.0	186 (172; 203)
Derived Management scenario	9.4	7.2	5.1	1,350	1,216	-134	8,214	8,223	9.0	S-SCORE dominates SCORE	6.1	304 (263; 359)
MR*	0.0	16.5	16.5	1,395	2,040	645	8,093	8,103	9.2	70,109	12.6	95 (76; 126)
Age 40-70*	7.0	13.6	11.8	1,425	1,657	232	8,174	8,184	10.2	22,745	11.0	140 (115; 179)
Male*	13.1	18.4	12.2	1,867	2,041	174	8,120	8,131	11.1	15,676	8.7	149 (114; 216)
Time Horizon 15y scenario	10.1	15.8		2,116	2,389	273	11,243	11,259	16.1	16,992	8.9	128 (119; 139)
MHR*	10.8	22.1	23.6	1,978	2,338	360	8,049	8,069	20.2	17,822	15.0	74 (63; 90)
Age 40-70, MHR*	8.6	20.7	22.0	1,915	2,305	390	8,050	8,070	20.3	19,212	15.2	72 (61; 87)
HR*	50.4	42.7	49.8	4,108	3,429	-679	7,888	7,948	60.3	S-SCORE dominates SCORE	21.8	42 (34; 54)
Age 40-70, HR*	50.7	41.9	49.7	4,423	3,585	-838	7,834	7,909	75.4	S-SCORE dominates SCORE	23.5	33 (27; 43)

Results for subgroups and scenarios sorted by increasing incremental effectiveness for S-SCORE vs. SCORE. Derived Management scenario (DM) refers to a base case scenario with management assumptions for S-SCORE assigned to the optimum values from univariate sensitivity analyses. MR: Moderate SCORE risk category. MHR: Moderate and high SCORE risk category. HR: High SCORE risk category. IC: Incremental costs (S-SCORE vs. SCORE). IE: Incremental Quality adjusted life years (S-SCORE vs. SCORE). ICER: Incremental cost-effectiveness ratio (IC/IE). RRR: Relative risk reduction compared to SCORE in the respective scenario. NNS: Number needed to screen to prevent one CVD event. All analyses followed the BC sampling approach except * which were based on microsimulation.

Fig S 4. Change in incremental net monetary benefit (INMB) by varying input assumptions.

Relative importance of variables from univariate sensitivity analyses by demonstrating the change in INMB of the upper bound value relative to the lower bound. Results from independent microsimulation analyses with 100,000 bootstrapped samples. INMB: Incremental net monetary benefit at a willingness-to-pay threshold of 50,000 €. With an increasing input value of the respective variable, a negative INMB indicates that S-SCORE is less preferred, while a positive INMB indicates a favorable effect for S-SCORE.

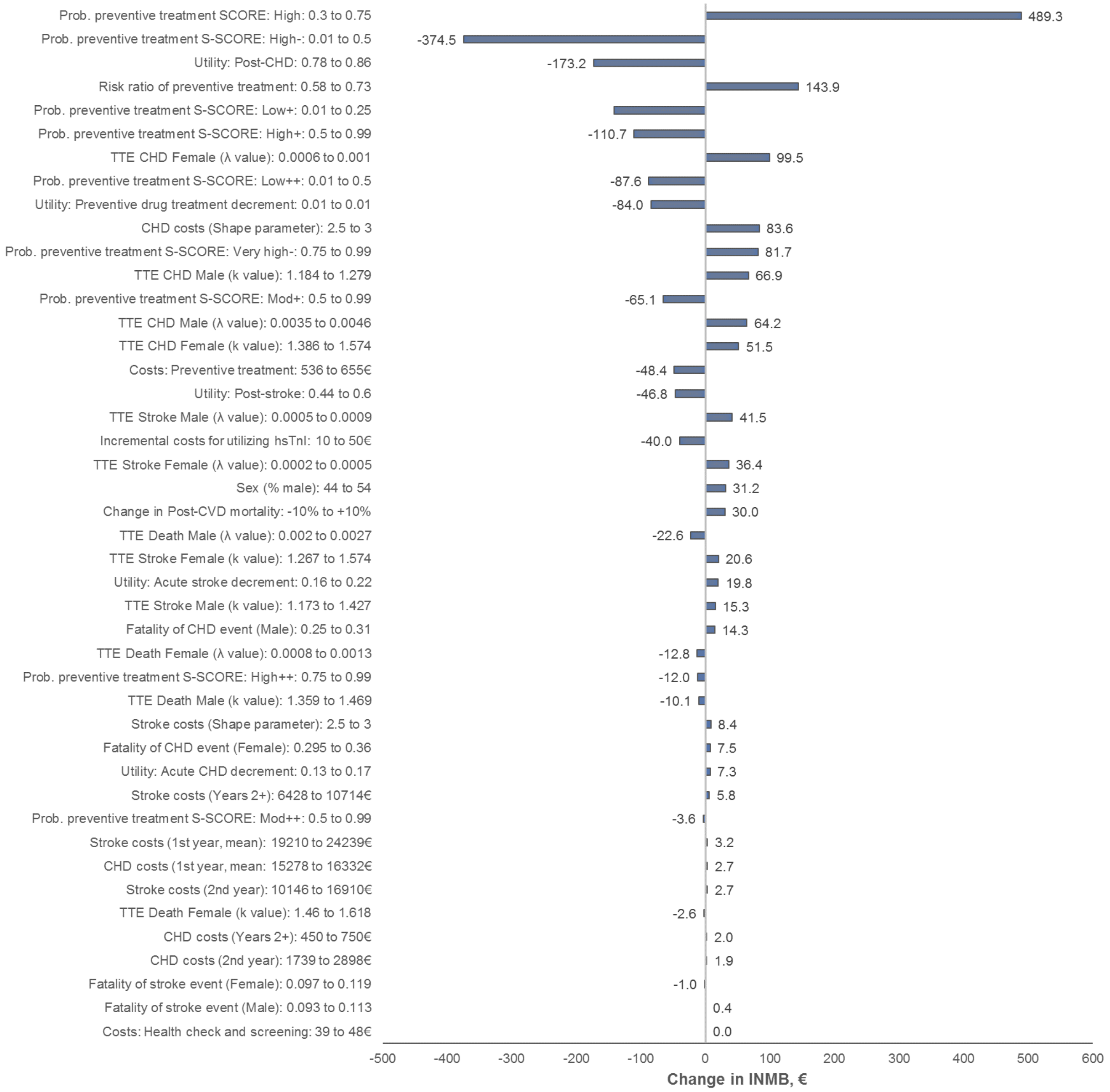

Fig S 5. Cumulative failure probabilities over 10-year follow-up.

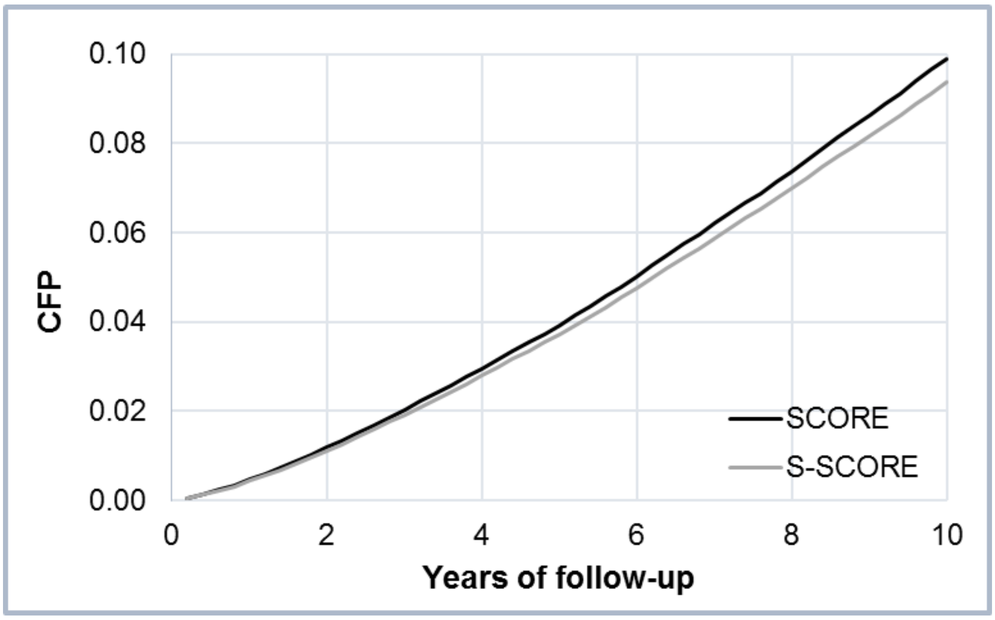

Cumulative failure probability (CFP) is defined as the combined likelihood of CVD events and death as observed in the study by each strategy.

References

1. Osler M, Linneberg A, Glümer C, Jørgensen T. The cohorts at the Research Centre for Prevention and Health, formerly 'The Glostrup Population Studies'. International Journal of Epidemiology. 2010;40(3):602-10.

2. Löwel H, Meisinger C, Heier M, Hörmann AJDG. The population-based acute myocardial infarction (AMI) registry of the MONICA/KORA study region of Augsburg. 2005;67(S 01):31-7.

3. Peltonen M, Lundberg V, Huhtasaari F, Asplund KJJoim. Marked improvement in survival after acute myocardial infarction in middle-aged men but not in women. The Northern Sweden MONICA study 1985–94. 2000;247(5):579-87.

4. Giampaoli S, Urbinati G, Menotti A, Ricci Gw, Pasquali MJEJoE. Short term changes in cardiovascular risk factors in the DI. S. CO. intervention project. 1991;7(4):372-9.

5. Di Castelnuovo A, De Curtis A, Costanzo S, Persichillo M, Olivieri M, Zito F, Donati MB, de Gaetano G, Iacoviello LJH. Association of D-dimer levels with all-cause mortality in a healthy adult population: findings from the MOLI-SANI study. 2013;98(9):1476-80.

6. Gianfagna F, Veronesi G, Guasti L, Chambless LE, Brambilla P, Corrao G, Mancia G, Cesana G, Ferrario MMJA. Do apolipoproteins improve coronary risk prediction in subjects with metabolic syndrome? Insights from the North Italian Brianza cohort study. 2014;236(1):175-81.

7. Borodulin K, Vartiainen E, Peltonen M, Jousilahti P, Juolevi A, Laatikainen T, Männistö S, Salomaa V, Sundvall J, Puska PJTEJoPH. Forty-year trends in cardiovascular risk factors in Finland. 2014;25(3):539-46.

8. Yarnell JJQmjotAoP. The PRIME study: classical risk factors do not explain the severalfold differences in risk of coronary heart disease between France and Northern Ireland. Prospective Epidemiological Study of Myocardial Infarction. 1998;91(10):667-76.

9. Tunstall-Pedoe H, Peters SA, Woodward M, Struthers AD, Belch JJJJotAHA. Twenty-year predictors of peripheral arterial disease compared with coronary heart disease in the Scottish Heart Health Extended Cohort (SHHEC). 2017;6(9):e005967.
